# Critical Analysis of Deconfounded Pretraining to Improve Visio-Linguistic Models

**DOI:** 10.3389/frai.2022.736791

**Published:** 2022-03-17

**Authors:** Nathan Cornille, Katrien Laenen, Marie-Francine Moens

**Affiliations:** Department of Computer Science, Language Intelligence and Information Retrieval (LIIR), KU Leuven, Leuven, Belgium

**Keywords:** causality, vision, language, deep learning, structural causal model (SCM)

## Abstract

An important problem with many current visio-linguistic models is that they often depend on spurious correlations. A typical example of a spurious correlation between two variables is one that is due to a third variable causing both (a “confounder”). Recent work has addressed this by adjusting for spurious correlations using a technique of deconfounding with automatically found confounders. We will refer to this technique as *AutoDeconfounding*. This article dives more deeply into *AutoDeconfounding*, and surfaces a number of issues of the original technique. First, we evaluate whether its implementation is actually equivalent to deconfounding. We provide an explicit explanation of the relation between *AutoDeconfounding* and the underlying causal model on which it implicitly operates, and show that additional assumptions are needed before the implementation of *AutoDeconfounding* can be equated to correct deconfounding. Inspired by this result, we perform ablation studies to verify to what extent the improvement on downstream visio-linguistic tasks reported by the works that implement *AutoDeconfounding* is due to *AutoDeconfounding*, and to what extent it is specifically due to the deconfounding aspect of *AutoDeconfounding*. We evaluate *AutoDeconfounding* in a way that isolates its effect, and no longer see the same improvement. We also show that tweaking *AutoDeconfounding* to be less related to deconfounding does not negatively affect performance on downstream visio-linguistic tasks. Furthermore, we create a human-labeled ground truth causality dataset for objects in a scene to empirically verify whether and how well confounders are found. We show that some models do indeed find more confounders than a random baseline, but also that finding more confounders is not correlated with performing better on downstream visio-linguistic tasks. Finally, we summarize the current limitations of *AutoDeconfounding* to solve the issue of spurious correlations and provide directions for the design of novel *AutoDeconfounding* methods that are aimed at overcoming these limitations.

## 1. Introduction

Recent years have seen great progress in vision and language research. Increasingly complex models trained on very large datasets of paired images and text seem to capture a lot of the correlations that are needed to solve tasks such as Visual Question Answering or Image Retrieval.

A concern however is that models make many of their predictions based on so-called *spurious correlations*: correlations that are present in the training data, but do not generalize to accurately make predictions when confronted with real world data.

To address this concern, researchers increasingly study the integration of causation into models. A model that is able to solve problems *in a causal way*, will be faster to adapt to other distributions and thus be more generalizable (Schölkopf, 2019). Because there are often multiple possible underlying causal models that each perfectly match the observed statistics, a key challenge is that it is not always easy to discover causal structure in purely observational data without being able to perform interventions^1^ on the data.

Recently, a technique has been proposed that aims to automatically discover and use knowledge of the underlying causal structure in order to avoid learning spurious correlations. More specifically, the goal is to avoid learning correlations that are due to a common cause (or “confounder”)^2^, a process called “deconfounding.” We will further refer to this technique as *AutoDeconfounding*. *AutoDeconfounding* was first developed by Wang et al. (2020) in their model named VC-R-CNN, and more recently adapted to the multi-modal setting by Zhang et al. (2020b) in their model DeVLBERT.

The goal of this article is to critically examine the technique of *AutoDeconfounding* and theoretically and empirically investigate whether *AutoDeconfounding* is an effective method to avoid spurious correlations. A closer inspection of *AutoDeconfounding* raises a number of questions that are addressed in this article.

First, deconfounding implies a certain assumption on the type of causal variables and their possible values. We make the underlying causal model of *AutoDeconfounding* explicit, and use that model to show that additional assumptions are needed before the implementation of *AutoDeconfounding* can be equated to correct deconfounding.

Inspired by this observation, we then set out to investigate to what extent the reported improvement on downstream tasks is due to *AutoDeconfounding*, and to what extent it is specifically due to the deconfounding aspect of *AutoDeconfounding*. Focusing on the most recent article (Zhang et al., 2020b), that implements *AutoDeconfounding* in a visio-linguistic context, we retrain and evaluate their model (“DeVLBERT-repro”) and their baseline (“ViLBERT-repro”) in a way that isolates the contribution of *AutoDeconfounding*. We compare the scores for our reproductions with the reported scores, as well as with the score of a pretrained checkpoint provided by Zhang et al. (2020b) (“DeVLBERT-CkptCopy.”) We also train and evaluate two newly created variations of DeVLBERT (“DeVLBERT-NoPrior” and “DeVLBERT-DepPrior”) intended to isolate the component within *AutoDeconfounding* that is hypothesized to be responsible for its beneficial effect. Our experiments show no noticable improvements of performance on downstream tasks with DeVLBERT-repro compared to ViLBERT-repro. Moreover, we show that DeVLBERT-NoPrior and DeVLBERT-DepPrior perform on-par with DeVLBERT-repro as well. This sheds doubt both on the role of deconfounding withing *AutoDeconfounding*, as on the effectiveness of *AutoDeconfounding* in general.

Finally, we investigate how accurately models that integrate *AutoDeconfounding* actually discover confounders. Such an experiment is relevant, because finding confounders from purely observational data seems to be at odds with the Causal Hierarchy Theorem (Bareinboim et al., 2020). For this purpose, we collect a human-labeled ground truth dataset of causal relations. We make two observations here. First, we find that DeVLBERT-repro and DeVLBERT-NoPrior outperform a random baseline in finding confounders, implying that *some* knowledge useful for identifying causes is present in the data. Second, we see no correlation between better confounder-finding and improved performance on downstream tasks: while DeVLBERT-CkptCopy and DeVLBERT-DepPrior score higher on downstream tasks, they are no better than a random baseline at finding confounders.

The contributions of this work are the following:

- We theoretically clarify what deconfounding in the visio-linguistic domain means, and show which additional assumptions need to be made for previous work to be equivalent to deconfounding.
- We verify the benefit of *AutoDeconfounding* on downstream task performance in a way that better isolates its effect, and fail to reproduce the reported gains.
- We collect a dataset of hand-labeled causality relations between object presence in visual scenes coming from the Conceptual Captions (Sharma et al., 2018) dataset. This dataset can be useful for validating future approaches to resolve spurious correlations through causality.

The rest of this article is structured as follows. In section 2, we discuss related work. In section 3, we explain what the terms causation, Structural Causal Models (SCMs)^3^ and deconfounding mean in the sense of the do-calculus of Pearl and Mackenzie (2018) and we explain how deconfounding could indeed theoretically improve performance on out-of-distribution downstream visio-linguistic tasks. In section 4, we show in detail how *AutoDeconfounding* is implemented in Zhang et al. (2020b) and Wang et al. (2020), and we explain what the various approximations they make mean in terms of assumptions on the underlying SCM. In section 5, we explain our methodology for investigating *AutoDeconfounding* more closely on three fronts: is its implementation equivalent to deconfounding, what explains its reported improvement on downstream visio-linguistic tasks, and are confounders found in its implementation. We discuss experimental results in section 6. Finally, we conclude in section 7.

## 2. Related Work

**Visio-linguistic models**. There has been a lot of work on creating the best possible general-purpose visio-linguistic models. Most of the recent models are based on the Transformer architecture (Vaswani et al., 2017), examples include ViLBERT (Lu et al., 2019), LXMERT (Tan and Bansal, 2019), Uniter (Chen et al., 2020), and VL-BERT (Su et al., 2019). Often, the Transformer architecture is complemented with a convolutional Region Proposal Network (RPN) to convert images into sets of region features: Ren et al. (2015) and Anderson et al. (2018), present examples of RPNs that have been used for this purpose. This articles that use *AutoDeconfounding*, which is the topic of this article, both use ViLBERT (Lu et al., 2019) as a basis for multi-modal tasks.

**Issue of spurious correlations**. The issue of models learning spurious correlations is widely recognized. Schölkopf et al. (2021) gives a good overview of the theoretical benefits of learning causal representations as a way to address spurious correlations. A number of works have tried to put ideas from causality in practice to address this issue. Most of these assume a certain fixed underlying SCM, and use this structure to adjust for confounders. Examples include Qi et al. (2020), Zhang et al. (2020a), Niu et al. (2021), or Yue et al. (2020). An important difference of *AutoDeconfounding* with regard to these works, is that in *AutoDeconfounding* the structure of the SCM is *automatically discovered*, as well as that the variables of the SCM correspond to individual object classes.

**Discovering causal structure**. There is theoretical work explaining the “ladder of causality” (Pearl and Mackenzie, 2018), where the different “rungs” of the ladder correspond to the availability of observational, interventional and counterfactual information, respectively. The Causal Hierarchy Theorem (CHT) (Bareinboim et al., 2020) shows that it is often very hard to discover the complete causal structure of an SCM (the second “rung” from the ladder) from purely observational data (the first “rung” of the ladder). However, it is not a problem for the CHT to discover the causal structure of an SCM up to its Markov Equivalence Class^4^. This has been done with constraint-based methods such as Colombo et al. (2012), and score-based methods such as Chickering (2002).

Despite the CHT, there have also been attempts to go beyond the Markov Equivalence class. One tactic to do this is through supervised training on ground truth causal annotations of synthetic data, and porting those results to real data (Lopez-Paz et al., 2017). Another way makes use of distribution-shifts to discover causal structure: this does not violate the CHT by being a proxy for having access to interventional (“second rung”) data. More specifically Bengio et al. (2019) and more recently Ke et al. (2020) train different models with different factorizations, see which model is the best at adapting to out-of-distribution data, and retroactively conclude which factorization is the “causal” one.

In contrast to these methods, *AutoDeconfounding* does not make use of distribution shifts nor of ground truth labeled causal data, but only of “first rung” observational data.

**Investigating** ***AutoDeconfounding***. The works that implement *AutoDeconfounding* (Wang et al., 2020 and Zhang et al., 2020b) both explain the benefit of *AutoDeconfounding* as coming from its deconfounding effect. This article will do novel additional experiments that surface a number of issues with *AutoDeconfounding*. We focus on the implementation by Zhang et al. (2020b) as it is the SOTA for *AutoDeconfounding*. First, we make the underlying SCM more explicit, showing the assumptions under which it corresponds to deconfounding. Second, we compare with the non-causal baseline in a way that better isolates the effect of *AutoDeconfounding*. Finally, we evaluate whether confounders (and thus “second rung” information about the underlying SCM) are indeed found by collecting and evaluating on a ground-truth confounder dataset.

## 3. Background: Causality

*AutoDeconfounding* is based on the do-calculus (Pearl, 2012), which is a calculus that operates on variables in so-called Structural Causal Models or SCMs. This section will explain the key aspects of SCMs and the do-calculus that are necessary to understand the discussion in the rest of this article.

### 3.1. Structural Causal Models

To understand SCMs, consider the following example. Say that we have observations of the presence (1) or absence (0) of rain clouds (*R*) and umbrellas (*U*) in a scene, see Table 1:

**Table 1:** Example observations of the presence of certain objects in a scene.

**Rain cloud (R)**	**Umbrella (U)**
1	1
0	0
0	0
1	0
⋮	⋮

We might observe the following joint probabilities: From



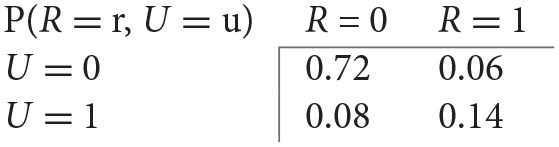



the point of view of causal modeling, any particular distribution of data observed by a model is generated by *physical, deterministic causal mechanisms* (Parascandolo et al., 2018).

For our example, say that the underlying mechanisms that generate the data are as follows. Whether or not it rains depends on factors outside of the observed data (such as the humidity of the air, the temperature, etc.). Whether or not an umbrella is present depends on whether it rains as well as on factors outside of the observed data (such as the psychology of the people carrying the umbrellas, etc.) Let us collect these external factors in the variables *E*_*R*_ for the rain cloud and *E*_*U*_ for the umbrella. Then, the value of *R* is determined by the function *R* = *f*_*R*_(*E*_*R*_), and the value of *U* is determined by the function *U* = *f*_*U*_(*R, E*_*U*_). Because the model cannot observe *E*_*R*_ and *E*_*U*_ however, the best it can do is view them as random variables, and try to learn their distribution.

In this way, the probability distributions of the *E*_*i*_, along with the causal mechanisms *f*_*i*_, generate a probability distribution of the observed variables: *P*(*R, U*). The set of observed variables and their relation is typically represented in an SCM. An SCM is a Directed Acyclic Graph (DAG) whose vertices consist of observed random variables *X, Y, Z*, … and in which a directed edge between nodes implies that the origin node is in the domain of the causal mechanism of the target node. Formally, if


(1) $$\begin{array}{l} X=f\left(PA_{X},E_{X}\right) \end{array}$$

then *PA*_*X*_ is the set of variables with outgoing arrows into *X*.

**Figure 1:**
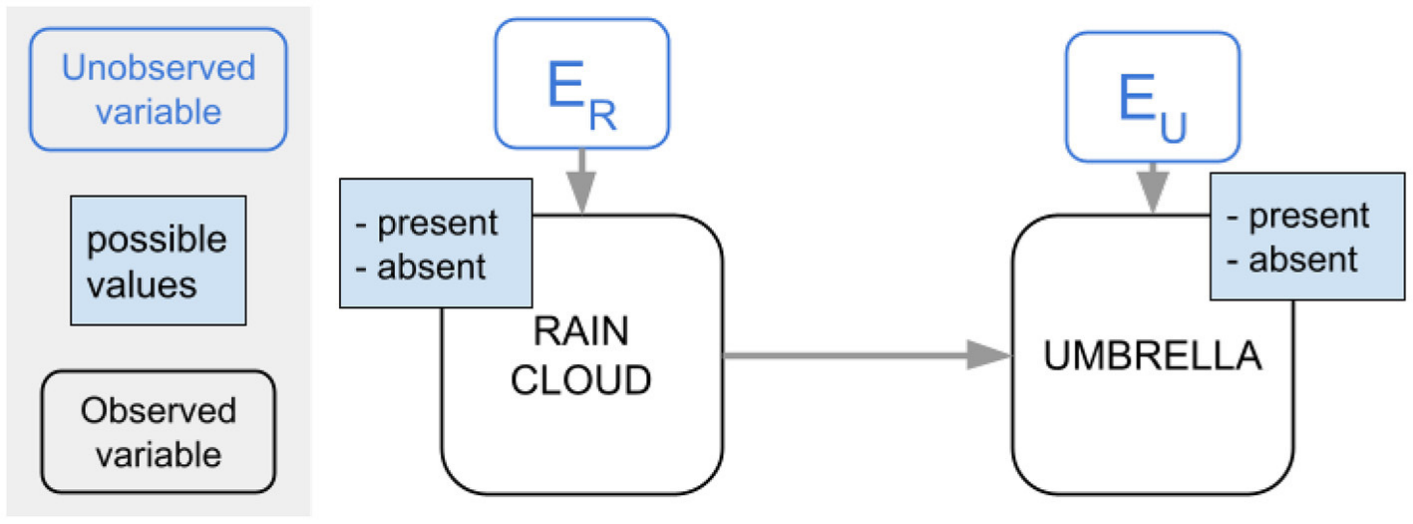
Example SCM.

Figure 1 shows the SCM for the example we discussed. Typically, the possible values and the unobserved variables are not explicitly shown, but we do so here for clarity.

### 3.2. Why Do We Want Models That Are More “Causal”?

To make the use of causality concrete for a visio-linguistic application, consider a model that needs to caption images. To do so successfully, it will need to recognize the different objects in the image. In order to recognize objects, the model will first of all make use of the per-object pixel-level information. However, to identify blurry, hard-to-recognize objects, it can complement that information with the context of the object. In other words, it can make use of knowledge about which objects are more or less likely to occur together. Consider the case where the image to be captioned is that of a rainy street. It is then useful for the model to be able to predict whether what it sees is a rain cloud given that it sees umbrellas (or vice versa).

A model that needs to learn parameters to perform this task can solve this in different ways. For instance, it could learn a parameter for each possible value of the joint probability *P*(*U, R*). This might be tractable for two variables, but for more variables, this approach requires too many parameters. Alternatively, it can learn a *factorization* of the joint probability and only learn parameters for each value of the factors. The two possible factorizations in this case are on the one hand *P*(*U, R*) = *P*(*U*|*R*)*P*(*R*), which is aligned with the underlying causal mechanism and on the other hand *P*(*U, R*) = *P*(*R*|*U*)*P*(*U*), which is not.

Each of these factorizations uses the same number of parameters, and each will be able to correctly answer queries like “what is the probability of not seeing umbrellas given that it rains.”

However, consider that there is a distribution change, for example, because we want our model to work for a location with more rain. In this case the factor *P*(*R*) has changed because of a change in the distribution of *E*_*R*_. To adapt, the factorization that was aligned with the underlying causal mechanism needs to change the parameter for only one factor, while the other factorization needs to change all its parameters.

Generalizing from this toy example, a distribution change typically only affects a few external factors *E*_*i*_. Because of this, a model with a factorization that is aligned with the underlying causal mechanisms (a “causal” factorization Schölkopf, 2019) will typically need to update fewer parameters than a model with another (“entangled”) factorization, and thus perform well on out-of-distribution data with fewer modifications (Bengio et al., 2019).

### 3.3. Do-Operator

Sometimes, we want to predict what *setting* the value of some variable *X* will have as an effect on the probability distribution of another variable *Y*, rather than what *observing* *X* will have as an effect.

Keeping Equation (1) in mind, “setting” a variable *X* to a value is the same as replacing the mechanism *X* = *f*(*PA*_*X*_, *E*_*X*_) that produces *X* with a constant *X* = *x*. Such an intervention to the underlying mechanisms then changes the resulting overall probability distribution. The distribution resulting from setting a variable *X* = *x* is indicated with the notation of the “do”-operator: the distribution of another variable *Y* in the SCM after we set (*X* = *x*) is noted as


(2) $$\begin{array}{l} P\left(Y|do\left(X=x\right)\right). \end{array}$$

Note the difference with the distribution of *Y* given that we *observe* *X* (rather than setting it):


(3) $$\begin{array}{l} P\left(Y|X=x\right). \end{array}$$

An important case where the do-operator highlights the difference between observation and intervention is in the case of confounders. When two variables *X* and *Y* share a common parent *Z* in the SCM, this parent is called a *confounder*. The statistical dependence between *X* and *Y* is then (at least partly) attributable to this parent.

In the presence of a confounder, *P*(*Y*|*X* = *x*) will be different from *P*(*Y*|*do*(*X* = *x*)).

**Figure 2:**
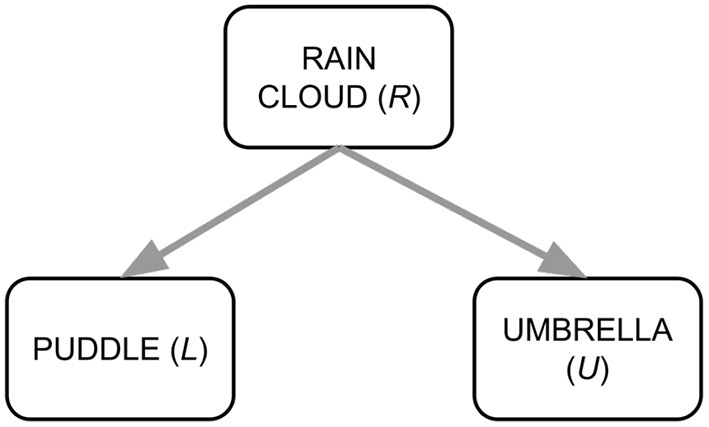
Example SCM.

For example, consider the SCM in Figure 2. We might reasonably expect *P*(*L* = 1|*U* = 1) > *P*(*L* = 1|*U* = 0): the probability that a puddle is present increases given that we *observe* an umbrella. On the other hand, we should not expect that the probability of a puddle being present changes when we *put* an umbrella in a scene: *P*(*L* = 1|*do*(*U* = 1)) = *P*(*L* = 1|*do*(*U* = 0)) = *P*(*L* = 1).

### 3.4. Deconfounding

The presence of a confounder can stand in the way of learning a causally aligned factorization. Consider again the SCM from Figure 2. The correct causal factorization is *P*(*R, L, U*) = *P*(*U*|*R*)*P*(*L*|*R*)*P*(*R*), but the correlation between *puddle* and *umbrella* might cause the model to use *P*(*L*|*U*) or *P*(*U*|*L*) as a factor. To discourage using these factors, we want to teach the model *P*(*L*|*do*(*U*)) (resp. *P*(*U*|*do*(*L*))) instead, as this spurious correlation disappears in the interventional view. In many domains however, such as the visio-linguistic domain, we cannot do actual real-life “interventions” on the data: we cannot “put” a rain cloud in a captioned image of a street and expect umbrellas to appear.

However, if we know the underlying SCM, we can still estimate *P*(*Y*|*do*(*X*)). To do so, we have to make sure that each confounder *Z*_*i*_ is adjusted for. Intuitively, we want each confounder to be “homogeneous” (Pearl, 2009) with regard to *X*: we should take samples from the SCM in such a way that each *Z*_*i*_ has the same distribution for every value of *X*. In this way, we “neutralize” any effect *Z*_*i*_ might have.

For the example of Figure 2, if we want to discover whether there is a causal link between *P* and *U*, we want to compare the difference in how many times we see puddles for the case in which there *is* an umbrella present, with the case in which there *is not*, without being confounded by the effect of one case having rain more often than the other case. We do this by making sure there are as many rainy days for the case without an umbrella as for the cases with an umbrella.

More generally, there can be more than one confounder (e.g., if there are also sprinklers that cause people to take out their umbrellas and puddles to form), and each variable can have more than 2 possible values (e.g., if the variable is “color of the umbrella” rather than “presence of an umbrella.”) In this more general case, the “back-door criterion” tells us which set of variables *S*_*Z*_ = {*Z*_1_, …, *Z*_*n*_} can be selected to be adjusted for to discover the causal link between two target variables *X* and *Y*: *S*_*Z*_ is any set of variables such that:

- No node in *S*_*Z*_ is a descendant of *X*;
- The nodes in *S*_*Z*_ block every path between *X* and *Y* that contains an arrow into *X*.

Note that this means it is also possible to adjust for *too many* variables, creating a spurious correlation where there was none before adjusting, so simply adjusting for *every* variable will not work.

Formally, if each *Z*_*i*_ ∈ *S*_*Z*_ has *n*_*i*_ possible values $v_{z_{i}}^{1},\ldots ,v_{z_{i}}^{n_{i}},i=1\ldots c$:


(4) $$\begin{array}{l} P\left(Y|do\left(X\right)\right)=\overset{v_{z_{1}}^{n_{1}},\ldots ,v_{z_{c}}^{n_{c}}}{\underset{v_{z_{1}}=v_{z_{1}}^{1},\ldots ,v_{z_{c}}=v_{z_{c}}^{1}}{\sum }}P\left(Y,Z_{1}=v_{z_{1}},\ldots ,Z_{c}=v_{z_{c}}|do\left(X\right)\right) \end{array}$$

(5) $$\begin{array}{l} =\overset{v_{z_{1}}^{n_{1}},\ldots ,v_{z_{c}}^{n_{c}}}{\underset{v_{z_{1}}=v_{z_{1}}^{1},\ldots ,v_{z_{c}}=v_{z_{c}}^{1}}{\sum }}P\left(Y|do\left(X\right),Z_{1}=v_{z_{1}},\ldots ,Z_{c}=v_{z_{c}}\right)\\ \cdot P\left(Z_{1}=v_{z_{1}},\ldots ,Z_{c}=v_{z_{c}}|do\left(X\right)\right) \end{array}$$

(6) $$\begin{array}{l} =\overset{v_{z_{1}}^{n_{1}},\ldots ,v_{z_{c}}^{n_{c}}}{\underset{v_{z_{1}}=v_{z_{1}}^{1},\ldots ,v_{z_{c}}=v_{z_{c}}^{1}}{\sum }}P\left(Y|X,Z_{1}=v_{z_{1}},\ldots ,Z_{c}=v_{z_{c}}\right)\\ \cdot P\left(Z_{1}=v_{z_{1}},\ldots ,Z_{c}=v_{z_{c}}\right) \end{array}$$

For example, for the case where there are only two confounders *Z*_1_ and *Z*_2_, each with only two possible values: absent (0) and present (1), Equation (6) becomes


(7) $$\begin{array}{l} P\left(Y|do\left(X\right)\right)=P\left(Y|X,Z_{1}=0,Z_{2}=0\right)\cdot P\left(Z_{1}=0,Z_{2}=0\right)+\\ \;P\left(Y|X,Z_{1}=0,Z_{2}=1\right)\cdot P\left(Z_{1}=0,Z_{2}=1\right)+\\ \;P\left(Y|X,Z_{1}=1,Z_{2}=0\right)\cdot P\left(Z_{1}=1,Z_{2}=0\right)+\\ \;P\left(Y|X,Z_{1}=1,Z_{2}=1\right)\cdot P\left(Z_{1}=1,Z_{2}=1\right) \end{array}$$

This example is relevant as it applies to *AutoDeconfounding*, where variables are different objects, and their possible values are either “present” (1) or “absent” (0). The SCM assumed by *AutoDeconfounding* does not put restrictions on the connectivity between objects, as long as the resulting graph is a DAG. Moreover, it assumes no hidden confounders.

We will discuss the link of *AutoDeconfounding* with deconfounding in more detail in section 5.1, after first clarifying the details of *AutoDeconfounding* itself in section 4.

## 4. Details of *AutoDeconfounding*

There are two variations of *AutoDeconfounding*: the one as implemented in VC-R-CNN (Wang et al., 2020), referred to as AD-V, and the one as implemented in DeVLBERT (Zhang et al., 2020b), referred to as AD-D.

Both Wang et al. (2020) and Zhang et al. (2020b) aim to improve on visio-linguistic tasks (e.g., Visual Question Answering or Image Captioning). Their models fall within a category of approaches that use *transfer learning*: They pretrain on a task different from the target task (a “proxy” task) for which more data is available (for example, context prediction or masked language modeling), and then fine-tune the resulting model on the actual downstream tasks of interest. Their innovation comes from an adaptation to the proxy task that is intended to prevent the model from learning spurious correlations.

VC-R-CNN and DeVLBERT differ slightly in both the context in which they use *AutoDeconfounding* and the exact implementation of *AutoDeconfounding*. This section will explain both models in detail.

### 4.1. VC-R-CNN

#### 4.1.1. Backbone and Modalities

The backbone of VC-R-CNN (Wang et al., 2020) is an image-region feature extractor [BUTD (Anderson et al., 2018)] which produces feature vectors for all regions-of-interest (ROIs) in an image. The image-region feature extractor is adapted by retraining it on a proxy task designed to prevent spurious correlations to be learned from vision data. Hence, VC-R-CNN focuses its contribution only on the image modality when pretraining.

#### 4.1.2. Proxy Task

During the pretraining, the loss function of VC-R-CNN consists of two terms:


(8) $$\begin{array}{l} L_{total,V}=L_{base,V}+L_{\text{AD-V}}. \end{array}$$

The base objective *L*_*base,V*_ is ROI classification, i.e., predicting the class of each of the *N* ROIs in the image. More precisely, if *x*_*i*_ is the index of the class of the *i*th ROI and ***p*** is the vector of predicted probabilities computed based on feature vector ***f***_***x***_ extracted for the *i*th ROI, then the base objective is:


(9) $$\begin{array}{l} L_{base,V}=\overset{N}{\underset{i=1}{\sum }}-log\left(p\left[x_{i}\right]\right). \end{array}$$

The additional objective *L*_AD-V_ regards context prediction, i.e., predicting the class of one ROI based on the features of a different ROI in the image. It does this for each pair of ROIs in the image and sums the resulting losses:


(10) $$\begin{array}{l} L_{AD-V}=\overset{N}{\underset{i=1}{\sum }}-log\left(p_{y}^{x}\left[y_{i}\right]\right), \end{array}$$

where $p_{y}^{x}$ is a probability distribution over the possible ROI classes for the *i*th ROI computed based on feature vector ***f***_***x***_ of the context ROI to use for that prediction, *y*_*i*_ is the index of the class of the *i*th ROI, and *N* is the number of ROIs in an image.

#### 4.1.3. AD-V

In order to predict the context in a “causal” way, VC-R-CNN introduces two elements that are gathered from the entire dataset: a confounder dictionary Z and prior probabilities $P_{Z}$.

The confounder dictionary Z is a set of *C* vectors, one per image class, where each Z[c] consists of the average ROI feature of all ROIs in all images belonging to class *c*. Given ***f***_***y***_ and ***f***_***x***_, where ***f***_***y***_ is the feature vector of the ROI whose class is to be predicted and ***f***_***x***_ is the feature vector of the context ROI to use for that prediction, VC-R-CNN computes a vector of attention scores **α** to select among Z those variables that are confounders for the classes corresponding to ***f***_***x***_ and ***f***_***y***_. More precisely, the attention is computed as the inner product between ***f***_***y***_ and Z after projection to a shared embedding space, and converted to a probability distribution using softmax:


(11) $$\begin{array}{l} \alpha \left[c\right]=\text{softmax}\left(\langle W_{z}Z\left[c\right],W_{x}f_{y}\rangle \right) \end{array}$$

where α[*c*] denotes the element at the *c*th index in vector **α**, 〈·, ·〉 denotes the inner product, and $W_{z},W_{x}\in {\mathbb{R}}^{D\times D}$ with *D* the dimension of feature representations. In section 5.3, we use this **α** to investigate whether confounders are actually found.

Next, the model retrieves a pooled vector ***f***_***z***_ by taking a sum of each of the *C* vectors in Z weighted by both the attention score α[*c*] and the prior probability from $P_{Z}$:


(12) $$\begin{array}{l} f_{z}=\overset{C}{\underset{c=1}{\sum }}Z\left[c\right]\alpha \left[c\right]P_{Z}\left[c\right]. \end{array}$$

Here, vector $P_{Z}$ is a probability distribution over the classes according to how many images they occur in^5^. The weighting of Z by its prior is intended to realize the deconfounding. In section 5.1, we explain the exact link with deconfounding.

**Figure 3:**
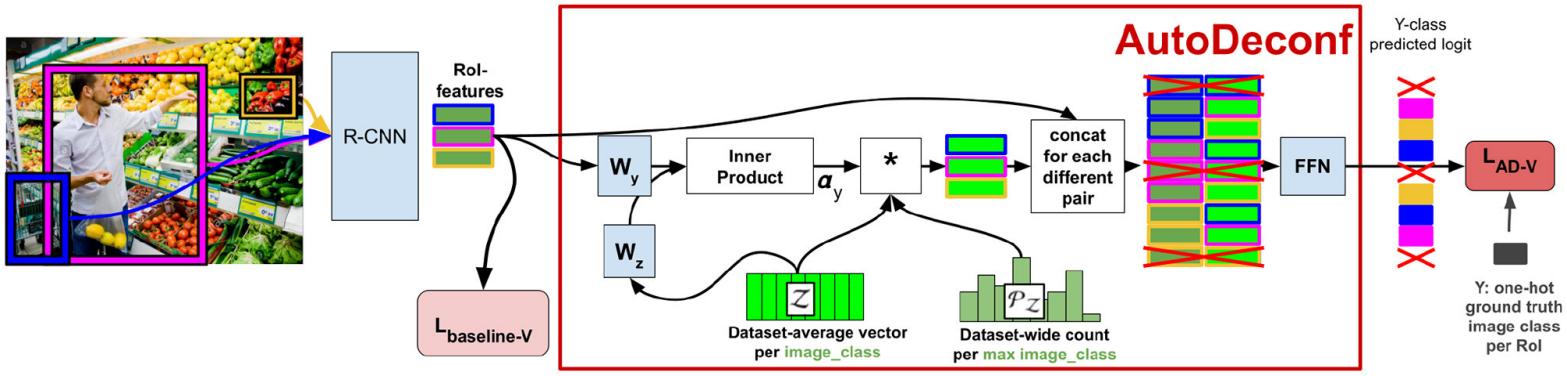
Pipeline for VC-R-CNN.

Finally, a simple feed-forward network *FFN* takes the concatenation of ***f***_***x***_ and ***f***_***z***_ and transforms it to the prediction over the possible classes $p_{y}^{x}$:


(13) $$\begin{array}{l} p_{y}^{x}=\text{softmax}\left(FFN\left(\left[f_{x};f_{z}\right]\right)\right), \end{array}$$

where [·;·] denotes the concatenation operation. The pipeline for VC-R-CNN is shown in Figure 3.

In order to clarify the analysis that we make in section 5.1, it is useful to rewrite $p_{y}^{x}$. First, in Equation (13) we can write the feed-forward network *FFN* as the function *g*_*V*_:


(14) $$\begin{array}{l} FFN\left(\left[f_{x};f_{z}\right]\right)=g_{V}\left(f_{x},f_{z}\right). \end{array}$$

Second, in Equation (12) assume that each α[*c*] were to perfectly select confounders (i.e., if there are *c* true confounders, give each of those *c* true confounders a weight of $\frac{1}{c}$^6^, and all other variables a weight of 0). Use *S*_*z*_ to denote the set of *c* indices of vectors in $P_{Z}$ for which the corresponding class is a confounder for the classes corresponding to ***f***_***x***_ and ***f***_***y***_. Then, we can summarize $p_{y}^{x}$ as:


(15) $$\begin{array}{l} p_{y}^{x}=\text{softmax}\left(g_{V}\left(f_{x},\underset{i\in S_{z}}{\sum }Z\left[i\right]\frac{1}{c}P_{Z}\left[i\right]\right]\right)) \end{array}$$

(16) $$\begin{array}{l} =\text{softmax}\left(g_{V}\left(f_{x},\frac{\sum_{i\in S_{z}}Z\left[i\right]P_{Z}\left[i\right]}{c}\right]\right)). \end{array}$$

Note that in the case where c > 1, the softmax from Equation (11) will tend to promote the selection of one confounder, even if there are multiple attention scores that are quite high in the input of the softmax.

To deal with the case of multiple confounders, a sigmoid function, subsequently scaled so that the entries still sum to one, would have been a better choice. However, as Zhang et al. (2020b) and Wang et al. (2020) use a softmax for *AutoDeconfounding*, we will consider the softmax in our further analysis.

#### 4.1.4. Pretraining Data

VC-R-CNN uses image datasets with ground truth bounding boxes [MS-COCO (Lin et al., 2014)] and Open Images Kuznetsova et al., 2020). The regions within these bounding boxes are the ROIs.

#### 4.1.5. Downstream Tasks

VC-R-CNN aims to improve performance on Image Captioning (IC), Visual Commonsense Reasoning (VCR) (Zellers et al., 2019) and Visual Question Answering (VQA) (Antol et al., 2015). More precisely, the image features extracted with VC-R-CNN are used as part of the pipeline of the various downstream models^7^.

### 4.2. DeVLBERT

#### 4.2.1. Backbone and Modalities

DeVLBERT (Zhang et al., 2020b) uses the exact same backbone and modalities as ViLBERT (Lu et al., 2019). Like ViLBERT, it uses a Faster R-CNN region extractor (Ren et al., 2015) [with ResNet-101 (He et al., 2016) backbone] to convert images into sets of region features, and initializes the weights for the linguistic stream with a BERT language model pretrained on the BookCorpus and English Wikipedia. It also adds the same cross-modal parameters.

Just like ViLBERT, DeVLBERT then performs visio-linguistic pretraining. The only difference is that it adds a number of “causal” parameters and losses during this pretraining, intended to make the model less prone to spurious correlations. These are detailed in the rest of this section. When the model is finetuned for downstream tasks, these extra parameters are no longer used: their only purpose was to change the “non-causal” parameters.

#### 4.2.2. Proxy Task

During the pretraining, the loss function for DeVLBERT is:


(17) $$\begin{array}{l} L_{total,D}=L_{base,D}+L_{\text{AD-D}}. \end{array}$$

DeVLBERT's base objective equals the one described in ViLBERT (Lu et al., 2019) which consists of a masked token modeling loss for each modality (*L*_*MTM*_*V*__ and *L*_*MTM*_*T*__), and a caption-image-alignment prediction loss (*L*_*VLA*_):


(18) $$\begin{array}{l} L_{base,D}=L_{VLA}+L_{MTM_{T}}+L_{MTM_{V}}. \end{array}$$

DeVLBERT's additional objective is similar to that of VC-R-CNN, but then extended to the multi-modal setting. More precisely, DeVLBERT predicts the class index *y*_*i*_ of the *i*th token (where “tokens” are words for the text modality, and ROIs for the vision modality) based on the contextualized feature ***f***_***y***_ for that same token. This means *L*_AD-D_ consists of 4 loss terms:


(19) $$\begin{array}{l} L_{AD-D}=L_{AD-D}^{t2t}+L_{AD-D}^{t2v}+L_{AD-D}^{v2t}+L_{AD-D}^{v2v}, \end{array}$$

where *t* stands for the text modality and *v* for the vision modality. Similar as in VC-R-CNN, a cross-entropy loss is used for the $L_{AD-D}^{\cdot 2\cdot }$ loss terms. For example, $L_{AD-D}^{t2v}$ is computed as:


(20) $$\begin{array}{l} L_{AD-D}^{t2v}=\overset{N}{\underset{i=1}{\sum }}-log\left(p_{y}^{t}\left[y_{i}^{t}\right]\right), \end{array}$$

where $y_{i}^{t}$ is the class index of the *i*th textual token, and the prediction over the possible classes $p_{y}^{t}$ for the *i*th textual token is computed using the confounder dictionary for the vision modality. The computation of the other $L_{AD-D}^{\cdot 2\cdot }$ loss terms is completely analogous.

#### 4.2.3. AD-D

**Figure 4:**
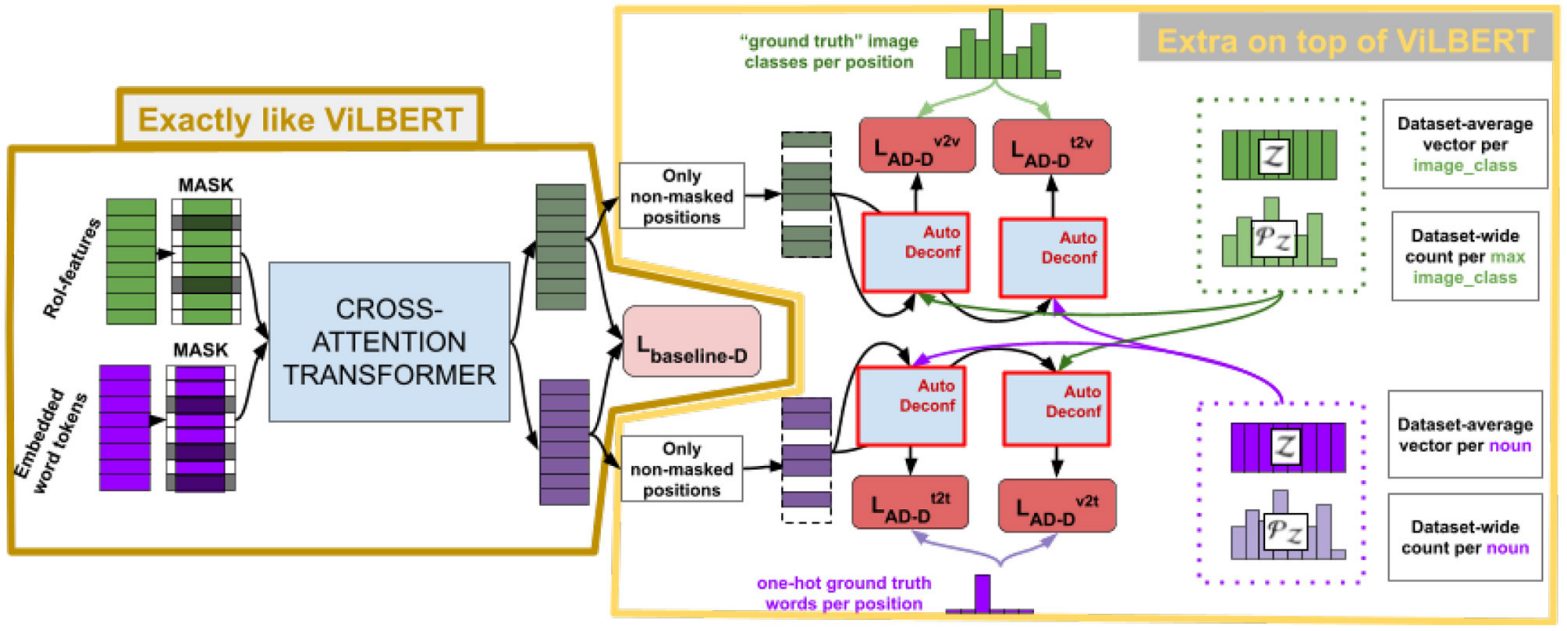
Pipeline for DeVLBERT.

The pipeline for DeVLBERT is shown in Figure 4.

Note that Figure 4 only describes variation “D” of the variations proposed in DeVLBERT, as this is the variation for which most results were reported in Zhang et al. (2020b).

Since DeVLBERT does *AutoDeconfounding* in the multi-modal setting, one of the main differences compared to VC-R-CNN is that modality-specific confounder dictionaries $Z^{t}$ and $Z^{v}$ and modality-specific prior probabilities ${P_{Z}}^{t}$ and ${P_{Z}}^{v}$ are used to do the context prediction in a “causal” way. In the following, we describe the pipeline for the text-to-vision case only, but the other cases are completely analogous.

In the text-to-vision case, for the context prediction of a textual token with feature vector $f_{y}^{t}$ the confounder dictionary for the visual modality $Z^{v}$ is used. First, attention scores **α** are calculated to select among the visual tokens in $Z^{v}$ those that are confounders for the textual token represented by $f_{y}^{t}$. Next, a pooled $f_{z}^{v}$ is calculated by weighting the *C* vectors in confounder dictionary Z based on the attention score α[*c*] and prior frequency ${P_{Z}}^{v}$:


(21) $$\begin{array}{l} \alpha \left[c\right]=\text{softmax}\left(\langle W_{z}Z^{v}\left[c\right],W_{r}f_{y}^{t}\rangle \right) \end{array}$$

(22) $$\begin{array}{l} f_{z}^{v}=\overset{C}{\underset{c=1}{\sum }}Z^{v}\left[c\right]\alpha \left[c\right]{P_{Z}}^{v}\left[c\right] \end{array}$$

where α[*c*] denotes the element at the *c*th index in vector **α**, 〈·, ·〉 denotes the inner product, and $W_{z},W_{r}\in {\mathbb{R}}^{D\times D}$ with *D* the dimension of feature representations. In section 5.1, we explain the exact link of weighting $Z^{v}$ by its prior ${P_{Z}}^{v}$ with deconfounding. Furthermore, in section 5.3, **α** is used to investigate whether confounders are actually found.

Another main difference with VC-R-CNN is the input for the prediction, which is not a concatenation, but only the pooled vector $f_{z}^{v}$:


(23) $$\begin{array}{l} p_{y}^{t}=\text{softmax}\left(FFN\left(f_{z}^{v}\right)\right). \end{array}$$

**Figure 5:**
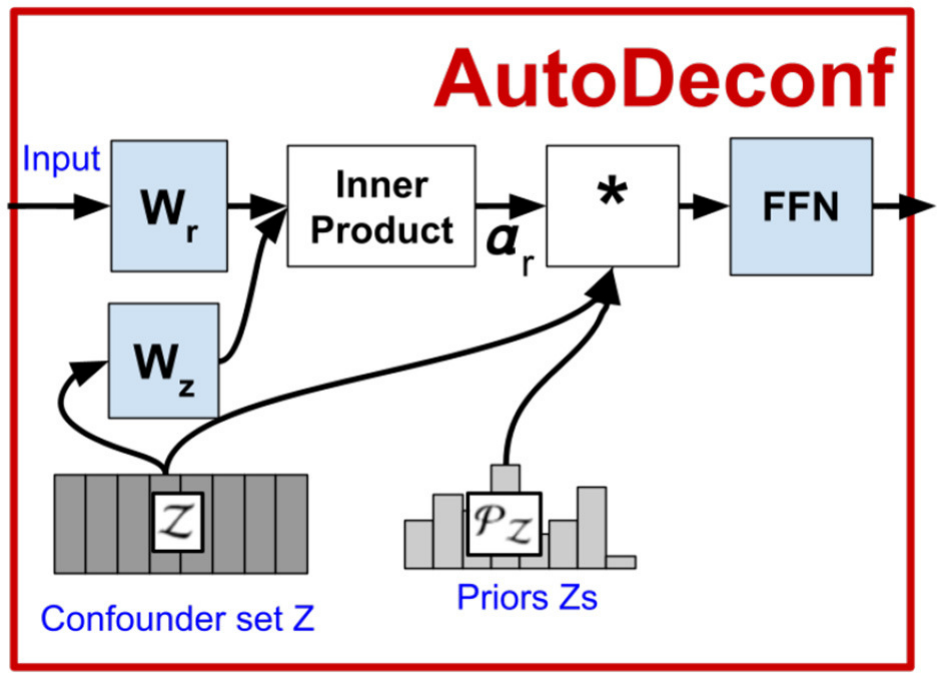
Zoom-in of AD-D.

**Figure 6:**
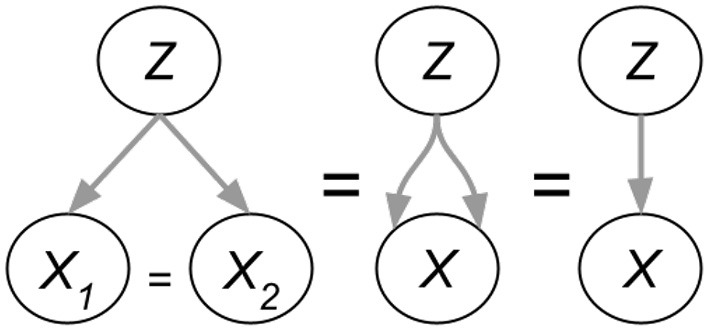
Three-way confounding relations collapsing to a two-way causal relation.

Figure 5 shows a zoom-in of the *AutoDeconfounding* operation in DeVLBERT (Equations 21–23). Note that for DeVLBERT, the ROI feature vector ***f***_***y***_ that is used to do the prediction (by selecting ***f***_***z***_ as an intermediate step), and the class *y*_*i*_ that we want to predict, actually correspond to the same token. In other words, DeVLBERT is finding variables that are confounders for the same variable. Zhang et al. (2020b) justify this by saying ***f***_***y***_ actually corresponds to a “mix” of tokens, since it has been contextualized through the self-attention mechanism in the ViLBERT backbone. However, this contextualization does not take away that ***f***_***y***_ mainly corresponds to the token in the image with class *y*_*i*_. In the three-way relation of a confounder, if the two variables *X*_1_ and *X*_2_ that are confounded by variable *Z* are one and the same (*X*_1_ = *X*_2_ = *X*), we can simply call *Z* a cause of *X*. Figure 6 illustrates this. We can thus also speak of “causes” instead of “confounders” when discussing DeVLBERT.

Again, we can rewrite $p_{y}^{t}$ into a form suitable for the analysis in section 5.1. First, we write the feed-forward network *FFN* as the function *g*_*D*_:


(24) $$\begin{array}{l} FFN\left(f_{z}^{v}\right)=g_{D}\left(f_{z}^{v}\right). \end{array}$$

Second, we simplify by assuming that each α[*c*] perfectly selects confounders (or in this case: causes) and that *S*_*z*_ is the set of *c* indices of vectors in ${P_{Z}}^{v}$ for which the corresponding class is a cause for the class corresponding to $f_{y}^{t}$. Then, we can summarize $p_{y}^{t}$ as:


(25) $$\begin{array}{l} p_{y}^{t}=\text{softmax}\left(g_{D}\left(\underset{i\in S_{z}}{\sum }Z^{v}\left[i\right]\frac{1}{c}{P_{Z}}^{v}\left[i\right]\right]\right)) \end{array}$$

(26) $$\begin{array}{l} =\text{softmax}\left(g_{D}\left(\frac{\sum_{i\in S_{z}}Z^{v}\left[i\right]{P_{Z}}^{v}\left[i\right]}{c}\right]\right)). \end{array}$$

#### 4.2.4. Pretraining Data

DeVLBERT uses a dataset of images paired with captions [Conceptual Captions (Sharma et al., 2018)]. We refer to an image-caption pair as a “record.” DeVLBERT does not use the images directly though, but uses a frozen region feature extraction network [BUTD (Anderson et al., 2018)] to represent the images as sets of ROI features. Note that for images, DeVLBERT does not use ground truth bounding boxes (as those do not exist for Conceptual Captions), but takes the predictions of the frozen and pretrained BUTD region proposal network as approximate ground truth.

#### 4.2.5. Downstream Tasks

For DeVLBERT, the downstream tasks are VQA, Image Retrieval (IR) and Zero-Shot Image Retrieval (ZSIR). Specifically, they train and evaluate VQA on the VQA 2.0 dataset (Antol et al., 2015) consisting of 1.1 million questions about COCO images (Chen et al., 2015), each with 10 correct answers, and (ZS)IR on the Flickr30k dataset (Young et al., 2014) consisting of 31 000 images from Flickr with five captions each. They exactly follow the splits and training setup of ViLBERT for this, as do we in the experiments described in the next section. Note that when applied to downstream tasks, DeVLBERT has the same architecture as ViLBERT: the only difference is that its weights are different due to the different pretraining objective (Equation 17).

#### 4.2.6. Out-Of-Distribution Setting

The DeVLBERT authors argue that there is a distribution shift between the pretraining data (Conceptual Captions) and the data of downstream tasks (VQA 2.0 and Flickr30k), namely because the captions in Conceptual Captions are automatically extracted based on alt-text attributes, whereas the VQA and Flickr30k captions are human-annotated. This is in line with other works on multi-modal pretraining that also consider Conceptual Captions to have no expected overlap with the data of common downstream tasks (Bugliarello et al., 2021). As explained in section 3.2, a model that makes prediction using only correlations that are also causal, should be expected to better adapt to a distribution shift.

Note that DeVLBERT uses the same region proposal network and text tokenizer for pretraining and downstream tasks. This means that the distribution is not different in *which* object or token classes appear, but rather in the statistics of the appearance of the same set of object or token classes.

To investigate the reported improvements of DeVLBERT, we copy their out-of-distribution setting. However, for future work, it can be interesting to consider a setting where the distribution shift is more explicit. For example, a distribution shift where certain classes are known to have different correlations.

## 5. Methodology

The previous sections provided background knowledge of causality and of *AutoDeconfounding*. This section will build upon that knowledge to examine *AutoDeconfounding* more closely.

Our methodology for investigating *AutoDeconfounding* consists of three parts: one theoretical analysis, and two empirical investigations for which we retrain a number of variations of the SOTA model that uses *AutoDeconfounding*. First, we theoretically examine whether the implementation of *AutoDeconfounding* actually corresponds to deconfounding. Second, we evaluate performance on downstream tasks to isolate which component of *AutoDeconfounding* is responsible for the reported improvements on those tasks. Finally, we examine to what extent confounders are actually found. We develop a ground truth dataset of causal relations to investigate this quantitatively, and qualitatively show a subset of the most-selected confounders.

### 5.1. Theoretical Examination of Deconfounding in *AutoDeconfounding*

The implementation of *AutoDeconfounding* was detailed in section 4. In this section, we explain how that implementation relates to the formula for a deconfounded prediction (Equation 6). We show the derivation as made in VC-R-CNN and DeVLBERT, and expand it to clarify the link with the underlying causal model.

The derivation starts with the formula for deconfounding:


(27) $$\begin{array}{l} P\left(Y|do\left(X\right)\right)=\underset{z}{\sum }P\left(Y|X,z\right)P\left(z\right) \end{array}$$

As explained in Equation 6, this is really a simplified notation for


(28) $$\begin{array}{l} P\left(Y\;=\;v_{y}|do\left(X\;=\;v_{x}\right)\right)\\ \;=\overset{1,\ldots ,1}{\underset{v_{z_{1}}\;=\;0,\ldots ,v_{z_{c}}\;=\;0}{\sum }}P\left(Y=v_{y}|X=v_{x},Z_{1}=v_{z_{1}},...,Z_{c}=v_{z_{c}}\right)\\ \;P\left(Z_{1}=v_{z_{1}},\ldots ,Z_{c}=v_{z_{c}}\right) \end{array}$$

where *v*_*x*_ and *v*_*y*_ each can be either 0, meaning “absent from the scene” or 1, meaning “present in the scene,” and {*Z*_1_…*Z*_*C*_} is the set of classes that form a confounder for *X* and *Y*. For simplicity of notation, we rewrite


$$\overset{1,\ldots ,1}{\underset{v_{z_{1}}=0,\ldots ,v_{z_{c}}=0}{\sum }}\langle \ldots \rangle P\left(Z_{1}=v_{z_{1}},\ldots ,Z_{c}=v_{z_{c}}\right)$$

as


$${𝔼}_{c_{z}}\left(\langle \ldots \rangle \right).$$

The derivation in VC-R-CNN and DeVLBERT then shows that *P*(*Y* = *v*_*y*_|*X* = *v*_*x*_, *Z*_1_ = *v*_*z*_1__, …, *Z*_*c*_ = *v*_*z*_*c*__) is calculated with softmax(*g*(***f***_***x***_, ***f***_***z***_)). Equation (28) then becomes:


(29) $$\begin{array}{l} P\left(Y=v_{y}|do\left(X=v_{x}\right)\right)={𝔼}_{c_{z}}\left(\text{softmax}\left(g\left(f_{x},f_{z}\right)\right)\right) \end{array}$$

Note that this implies that the state of *X* is encoded in ***f***_***x***_, and the joint state of *Z*_1_, …, *Z*_*c*_ is encoded in ***f***_***z***_.

The derivation then makes an approximation to move *E*_*c*_*z*__ into the softmax following the idea of the Normalized Weighted Geometric Mean, or NWGM. The idea of NWGM is similar to that of how dropout approximates an ensemble of models. It approximates the aggregate result of resampling (in multiple passes) cases where *X* = *v*_*x*_ so that *Z* occurs at the rate *P*(*Z* = *v*_*z*_) by 1) only doing one pass for *X* = *v*_*x*_, but 2) using the NWGM of the possible values *v*_*z*_ of *Z*, weighted by their prior distribution *P*(*Z* = *v*_*z*_).

Further, given that the function *g*(·, ·) is linear, the expectation can be moved next to the argument ***f***_***z***_:


(30) $$\begin{array}{ll} P\left(Y=v_{y}|do\left(X=v_{x}\right)\right)\; & \overset{\text{NWGM}}{\approx }\text{ softmax}\left({𝔼}_{c_{z}}\left(g\left(f_{x},f_{z}\right)\right)\right) \end{array}$$

(31) $$\begin{array}{l} =\text{softmax}(g\left(f_{x},{𝔼}_{c_{z}}\left(f_{z}\right)\right) \end{array}$$

Writing 𝔼_*c*_*z*__ in full again:


(32) $$\begin{array}{l} P\left(Y=v_{y}|do\left(X=v_{x}\right)\right)=\text{softmax}(g(f_{x},\overset{1,\ldots ,1}{\underset{v_{z_{1}}=0,\ldots ,v_{z_{c}}=0}{\sum }}\\ \;f_{z}P\left(Z_{1}=v_{z_{1}},\ldots ,Z_{c}=v_{z_{c}}\right)), \end{array}$$

we can now compare this with the actual implementation as explained in section 4 in Equations (16) and (26).


(33) $$\begin{array}{l} \text{VC-R-CNN:}p_{y}^{x}=\text{softmax}\left(g_{V}\left(f_{x},\frac{\sum_{i\in S_{z}}Z\left[i\right]P_{Z}\left[i\right]}{c}\right]\right)) \end{array}$$

(34) $$\begin{array}{l} \text{DeVLBERT:}p_{y}^{a}=\text{softmax}\left(g_{D}\left(\frac{\sum_{i\in S_{z}}Z^{b}\left[i\right]{P_{Z}}^{b}\left[i\right]}{c}\right]\right)) \end{array}$$

First, specifically for DeVLBERT (Equation 34), there is a mismatch in the first argument of *g*: ***f***_***x***_ is no longer involved for *g*_*D*_, meaning that the prediction is made based on *only* the state of the confounder variables.

Second, for both VC-R-CNN and DeVLBERT, there is an apparent mismatch in the second argument of *g*. Equation (32) shows that the sum should be taken over all possible combination of values for every possible confounder. However, in the actual implementation, Equations (33) and (34) show that the there is only one term per confounder, corresponding to the case where its value is “present.”

For example, if *Y* is puddle, *X* is umbrella, *Z*_1_ is rain cloud and *Z*_2_ is sprinkler, then there are two confounders for *P*(*Y*|*X*): *Z*_1_ and *Z*_2_. Instead of calculating *P*(*Y*|*do*(*X*)) by taking the average value of *P*(*Y*|*X, Z*_1_, *Z*_2_) weighted by the prior probabilities *P*(*Z*_1_ = *present, Z*_2_ = *present*), *P*(*Z*_1_ = *present, Z*_2_ = *absent*), *P*(*Z*_1_ = *absent, Z*_2_ = *present*), *P*(*Z*_1_ = *absent, Z*_2_ = *absent*), *AutoDeconfounding* takes the average value weighted by the prior probabilities *P*(*Z*_1_ = *present*), *P*(*Z*_2_ = *present*).

Despite this apparent mismatch, with two additional assumptions that were not specified in Zhang et al. (2020b) or Wang et al. (2020), it *is* possible for this second argument of *g* from Equation 32 to simplify into matching the second argument of *g* in Equations (33) and (34):

For the first assumption, note that in the implementation, the joint state *Z*_1_, …, *Z*_*c*_ is encoded as follows: For each *Z*_*i*_, if its state is “present,” it is represented by the average ROI feature vector ***f***_***z***_***i***__ of the corresponding class. These average vectors are the ones collected in the confounder dictionary Z. If its state is “absent,” it is represented by a zero-vector with the same shape as ***f***_***z***_***i***__. Then, the joint state is represented as the average vector $f_{z}=\frac{\sum_{i=1}^{c}{f_{z}}_{i}}{c}$. The assumption is then that ***f***_***z***_ can successfully distinguish all possible joint states.

The second assumption is that all the confounders are independent of one another. This entails that $P\left(Z_{1}=v_{z_{1}},\ldots ,Z_{c}=v_{z_{c}}\right)=\prod_{i=1}^{c}P\left(Z_{i}=v_{z_{i}}\right)$.

Under these two assumptions, it can be shown that terms of the sum in Equation (32) reduce (barring scaling factors) to the terms in the implementation Equations (33) and (34). The proof for this is in Appendix section 1.1.

In conclusion, we show that certain additional assumptions are needed to overcome the mismatch between theoretical deconfounding on the one hand, and the implementation of *AutoDeconfounding* on the other hand. More specifically, it is necessary to assume that the various confounders are independent of one another, and that the encoding of the joint confounder state as implemented in *AutoDeconfounding* can uniquely determine each state.

In the next sections, we evaluate aspects of *AutoDeconfounding* empirically. We perform our empirical experiments only for DeVLBERT, as it is the state-of-the-art of this articles that use *AutoDeconfounding*.

### 5.2. Ablation Studies on Downstream Performance

As explained in section 4.2, Zhang et al. (2020b) evaluate the quality of the DeVLBERT model by its performance on downstream visio-linguistic tasks. They report a significant improvement on these tasks when extending their baseline model with *AutoDeconfounding*.

Our analysis from section 5.1 shows that additional assumptions are necessary to equate the implementation of *AutoDeconfounding* with deconfounding. Inspired by this, we perform ablation studies to verify to what extent deconfounding is actually responsible for the reported improvement in scores. We do this by adapting *AutoDeconfounding* in such a way that it retains access to the confounder dictionary, but is no longer related to deconfounding. We also verify the extent of the contribution of *AutoDeconfounding* as a whole, by comparing with a baseline that does not use *AutoDeconfounding* on a more like-for-like basis.

Because we want to investigate the relation between the implementation of *AutoDeconfounding* and the performance increase on downstream tasks as reported by DeVLBERT, we evaluate on exactly the same downstream tasks as DeVLBERT.

#### Isolating the Contribution of the Confounder Dictionary

For our first experiment on downstream task performance, we test the hypothesis that the key ingredient is the use of the “confounder” dictionary Z. We hypothesize that Z forces contextualized representations to be sufficiently close to a per-class average representation, thus providing some kind of regularization. In this hypothesis, $P_{Z}$ is an irrelevant component of *AutoDeconfounding*. To isolate the effect of Z, we have implemented and trained-from-scratch two variations of *AutoDeconfounding* for which we alter $P_{Z}$.

First, we create a model named DeVLBERT-NoPrior in which $P_{Z}$ is left out completely. This changes Equation (22) to:


(35) $$\begin{array}{l} f_{z}^{b}=\overset{C}{\underset{c=1}{\sum }}\alpha \left[c\right]\cdot 1\cdot Z^{b}\left[c\right]. \end{array}$$

Second, we create a model named DeVLBERT-DepPrior in which $P_{Z}$ is replaced by a dependent prior. As opposed to vanilla DeVLBERT, DeVLBERT-DepPrior does not weight each entry in Z by the prior frequency of the class corresponding to that entry. Rather, it takes into consideration both the class *C*_*z*_ of the entry in the confounder dictionary Z, and the class *C*_*y*_ of the token that is being predicted. It weights each entry by the frequency of tokens of class *C*_*z*_ *within* records of the Conceptual Captions dataset where a token of class *C*_*y*_ is also present.

Because DeVLBERT has a loss term for each combination of modalities, DeVLBERT-DepPrior has a dependent prior for each modality combination. For example, the dependent prior ${P_{ZD}}^{t2v}$ used to calculate the loss term $L_{AD-D}^{t2v}$ is:


(36) $$\begin{array}{l} {P_{ZD}}^{t2v}\left[j,k\right]=\frac{J_{t,v}\left[j,k\right]}{M_{t}\left[j\right]} \end{array}$$

(37) $$\begin{array}{l} J_{t,v}\left[j,k\right]=\overset{T}{\underset{i=1}{\sum }}I_{t}\left(i,j\right)\cdot I_{v}\left(i,k\right) \end{array}$$

(38) $$\begin{array}{l} M_{t}\left[j\right]=\overset{T}{\underset{i=1}{\sum }}I_{t}\left(i,j\right) \end{array}$$

Where *T* is the total number of records, *j* and *k* denote the class indexes for modality *t* and *v*, respectively, and *I*_*t*_(*j, k*) is an indicator function that is 1 if modality *t* of the *j*th record contains a token with class index *k* and 0 otherwise.

Equation (22) then becomes:


(39) $$\begin{array}{l} f_{z}^{v}=\overset{C}{\underset{c=1}{\sum }}\alpha \left[c\right]\cdot {P_{ZD}}^{t2v}\left[i,c\right]\cdot Z^{v}\left[c\right]. \end{array}$$

where *i* is the index of *C*_*y*_ and *c* the index of *C*_*z*_.

If these variations achieve a similar score to DeVLBERT, that supports the hypothesis that Z is the key component of *AutoDeconfounding*.

#### Comparing Like-For-Like

For our second experiment on downstream task performance, we observe that the comparison between DeVLBERT and ViLBERT in Zhang et al. (2020b) is not made on a completely like-for-like basis, and so does not properly isolate the effect of *AutoDeconfounding*.

More specifically, DeVLBERT was trained for 24 epochs, where for the last 12 epochs the region mask probability is changed from 0.15 to 0.3 (Zhang et al., 2020b), whereas ViLBERT is only trained for 10 epochs (with region mask probability 0.15)(Lu et al., 2019). Zhang et al. (2020b) report that longer pretraining can be especially beneficial for zero-shot IR performance^8^. Moreover, for fine-tuning, ViLBERT uses the last checkpoint for evaluation, whereas DeVLBERT uses the best checkpoint (based on the validation score) for evaluation.

We retrain and ViLBERT and DeVLBERT ourselves on a like-for-like basis. The details of our experimental setup are in section 6.1.

### 5.3. Investigating Confounder Finding

The last question we investigate is whether confounders are actually found. According to the Causal Hierarchy Theorem (Bareinboim et al., 2020), with access to only observational data, you cannot make a model that correctly answers interventional (causal) queries for “almost-all^9^” underlying SCMs. Or as stated by Cartwright (1994): “no causes-in, no causes-out.” Moreover, Zhang et al. (2020b) and Wang et al. (2020) did not quantitatively verify whether confounders are found, but take this as a given. Taking the above into account, it is not obvious that confounders are actually found by *AutoDeconfounding*.

We evaluate the confounder-finding capacities of DeVLBERT both quantitatively and qualitatively. Because we focus on DeVLBERT, we will speak of “causes” instead of “confounders.”

A lot of the tokens from the text modality are not meaningful as causal variables (words such as “over,” “the,” etc.) Hence, we focus specifically on the image modality, where all of the tokens correspond to real objects.

#### 5.3.1. Quantitative Analysis

##### 5.3.1.1. How to Collect Ground Truth Confounders?

To check *quantitatively* whether actual causes are found, we need ground truth labels on the causality between objects in a scene.

To do this, we create a novel dataset with ground truth labels. Ideally, the way to gather causal labels would be to do interventions in the real world. However, this is difficult to realize (e.g., it is hard to “put” a rain cloud into a scene). Because many causal relations between objects are obvious to human common sense, we rely on human judgement to annotate causal relations instead. The assumption here is that the “mental intervention” that humans do when they answer a question like “Would changing the presence of ‘umbrella’ influence the presence of ‘rain cloud’ in the scene?” is a good approximation of the real-world intervention.

##### 5.3.1.2. Details of the Data Collection

*Selecting Data to Label* DeVLBERT works with 1,600 visual object classes, so the number of class-pairs for which a causal-link question can be asked is 1, 600^2^ = 2.56 million pairs. Because we believe most of these pairs will have no direct link at all, and labeling all 2.56 million pairs is too expensive, we select a subset of 1,000 class pairs to label. To select this subset, we use the following heuristic:

We assume that candidate pairs that are not correlated in the dataset, will not exhibit a causal link either^10^. Hence, to select a subset of pairs, we ranked pairs by how strongly they were correlated in the dataset. More specifically, if *P*(*X* = 1) is the probability that an image contains an object of class *X*, and *P*(*X* = 1, *Y* = 1) is the probability that an image contains both an object of class *X* and an object of class *Y*, we select classes for which the difference between *P*(*X* = 1, *Y* = 1) and *P*(*X* = 1)*P*(*Y* = 1) is large.

We select 500 class pairs for which the absolute difference |*P*(*X* = 1, *Y* = 1)−*P*(*X* = 1)*P*(*Y* = 1)| is the highest, and 500 pairs for which the relative difference *log*(*P*(*X* = 1, *Y* = 1)/*P*(*X* = 1)*P*(*Y* = 1))^11^ is the highest^12^.

**Assuring Label Quality**. To label the data, we used Amazon Mechanical Turk (MTurk)^13^. We ask workers to label pairs (*X*,*Y*) of correlated objects with one of three options: *X* causes *Y*, *Y* causes *X*, or neither (in other words some confounder *Z* causes *X* and *Y*). In the latter case, we also provide a free-form box where workers could enter what they thought this confounder would be. An example of the form we used can be found in the Supplementary Material. We also let workers fill in a confidence score of 1–3 of how confident they are in their answer.

The kind of causality that we target is non-trivial to non-experts: we say that one object “is the cause of” another if intervening on its presence influences the probability of the other object being present. To ensure that workers understand the task well, we provide detailed instructions in the form, along with an explanation video. We also require that workers get a minimum score on a test with a small number of pairs with an obvious causal direction. The test questions can be found in the Supplementary Material.

We let each pair be labeled by 5 different workers and keep only those pairs for which agreement was at least 4 out of 5^14^. This left us with 595 pairs (or about 60 percent of pairs with at least 4/5 agreement). Table 2 shows 10 of these pairs.

**Table 2:** Subset of response pairs from crowdworkers.

**Object 1 (X)**	**Object 2 (Y)**	**Most selected response**
Trick	skater	Y causes X
Laptops	office	Y causes X
Person	shirt	X causes Y
Table	man	a confounder Z causes X and Y
Face	tree	a confounder Z causes X and Y
Sleeve	shirt	Y causes X
Arm	man	Y causes X
Players	plant	a confounder Z causes X and Y
Nose	sky	a confounder Z causes X and Y

Table 3 shows some examples of confounders that workers entered in the free-form box. One pattern to observe is that when the two objects are parts of a whole, the whole is sometimes suggested (e.g., *windshield wipers* and *doors* are caused by a *car*, or *outfield* and *mound* by *baseball field*). Suggested confounders can also vary quite widely (e.g., *table, office*, or *coffee shop* as confounder for *coffee cup* and *monitors*).

**Table 3:** Subset of free-from confounders suggested by crowdworkers.

**Object 1 (X)**	**Object 2 (Y)**	**Free-form suggested confounders**
Hand	shoe	human, person, child
Ski poles	ski boot	mountains, skier, snow
Windshield wipers	doors	car, cars
Outfield	mound	baseball field, baseball
Barricade	cones	street, road, construction
Coffee cup	monitors	table, office, coffee shop
Cucumber	cauliflower	salad
Wall	pillow	bedroom, room
Geese	ducks	lake, animals

*Note that these were not used in the confounder ranking metric*.

The full dataset with responses, confidences and free-text confounder responses is publicly available on Google Drive^15^.

#### 5.3.1.3. Confounder Ranking Metric

Recall from Equation 22 that the mechanism by which causes are found consists of using attention scores **α** to pool vectors from Z whose classes correspond to causes.

We take the trained model from DeVLBERT, and for every ROI *y* in every image, we produce **α**. We then examine the objects classes *o*_*i*_ in our dataset *for which we have the ground truth relation to* *y* (i.e., either *o*_*i*_ is a cause of *y*, *y* is a cause of *o*_*i*_, or they are “mere correlates.”) We call the set of *o*_*i*_ for a particular *y* *O*_*y*_. A successful **α** should rank the *o*_*i*_ that are a cause of *y* higher than the *o*_*i*_ that are either consequences or mere correlates. We use mean average precision (mAP) as metric to measure the ranking performance. We compare the resulting mAP score with a baseline that ranks the elements of *O*_*y*_ in a completely random way.

We report on the results in section 6.2.1.

#### 5.3.2. Qualitative Analysis

The quantitative analysis only considers classes for which we have collected ground truth causality information. It can be informative, however, to look at the *complete* ranking of candidate causes for a certain class.

We calculate the per-class average value of α[*c*] over all *R* ROIs in the dataset whose ROI class corresponds to *c* for each class *c*:


(40) $$\begin{array}{l} \alpha_{avg}\left[c\right]=\frac{\sum_{i=1}^{R}I\left(i,c\right)\cdot \alpha \left[c\right]}{N} \end{array}$$

(41) $$\begin{array}{l} \alpha \left[c\right]=\langle \left(W_{z}Z^{I}\left[c\right]\right),\left(W_{x}f_{x}^{i}\right)\rangle \end{array}$$

where $f_{x}^{i}$ is the contextualized ROI-feature corresponding to the *i*th ROI, $Z^{I}$ is the confounder dictionary for the image modality, and *I*(*i, c*) is an indicator function that is 1 if the *i*th ROI has class *c* and 0 otherwise.

We also show a few qualitative examples during a downstream task, specifically VQA. We check the last-layer cross-modal attention from a query word in the question to the bounding box of an object which we know is a cause of the query word. This can indicate whether certain models pay more attention to actual causes during downstream tasks.

## 6. Results

### 6.1. Ablation Studies on Downstream Performance

#### Setup

As explained in section 4.2, Zhang et al. (2020b) evaluate the quality of the DeVLBERT model by its performance on three downstream visio-linguistic tasks: Image Retrieval and Visual Question Answering, for which the model is further fine-tuned, and Zero-Shot Image Retrieval, for which the pretrained model is immediately used. Performance on (Zero-Shot) Image Retrieval is measured by recall at *k* or *R@k*, and performance on Visual Question Answering is measured by accuracy. We evaluate in the same way.

When reproducing DeVLBERT, we have tried to follow the original setup as closely as possible. We train all models with the same batch size and learning rate as DeVLBERT^16^.

There are, however, still a few differences in set-up. First, for Visual Question Answering, we only report performance on the “test-dev” split, and not on the “test-std” split: only 5 submissions to “test-std” are allowed, and we evaluate more than 5 models. Second, because the Conceptual Captions dataset consists of hyperlinks to web content, over time some of the links go stale. Hence we work with 2.9 million records, compared to the 3.1 million that DeVLBERT originally trained on.

Finishing 24 epochs of pretraining on the Conceptual Captions dataset takes 3–5 days^17^.

A pretrained checkpoint was made publicly available by Zhang et al. (2020b). We redo only the fine-tuning step ourselves for this checkpoint, and also include its performance in our results. We refer to this run as DeVLBERT-CkptCopy.

To make sure that the improvement is indeed due to *AutoDeconfounding*, we retrain ViLBERT and DeVLBERT ourselves, this time in exactly the same way: both for 24 epochs, where for the last 12 epochs the region mask probability is changed from 0.15 to 0.3, and both using the best checkpoint in the fine-tuning tasks for evaluation.

#### Results

Table 4 shows an overview of the downstream tasks performances of the different models we evaluated^18^.

**Table 4:** Scores on downstream tasks: top-1 recall, top-5 recall and top-10 recall for Image Retrieval (IR R@1, IR R@5, IR R@10) and zero-shot image retrieval (ZSIR R@1, ZSIR R@5, ZSIR R@10), and accuracy on the test-dev split for Visual Question Answering (VQA).

	**IR R@1**	**IR R@5**	**IR R@10**	**ZSIR R@1**	**ZSIR R@5**	**ZSIR R@10**	**VQA test-dev**
**Run name**							
DeVLBERT reported	61.60	87.10	92.60	36.00	67.10	78.30	71.50
ViLBERT reported	58.20	84.90	91.50	31.90	61.10	72.80	70.90
DeVLBERT repro (5 run avg ± stdev)	61.06 ± 1.04	87.27 ± 0.48	92.78 ± 0.4	35.19 ± 0.97	64.56 ± 0.82	75.16 ± 0.65	71.05 ± 0.06
ViLBERT repro (5 run avg ± stdev)	62.13 ± 0.61	87.48 ± 0.43	92.92 ± 0.31	34.2 ± 1.17	63.53 ± 1.2	74.59 ± 1.25	70.45 ± 0.42
DeVLBERT-CkptCopy	61.56	87.56	93.02	37.42	66.74	77.88	70.88
DeVLBERT-DepPrior	63.70	87.86	93.20	32.40	62.70	73.80	70.29
DeVLBERT-NoPrior	61.12	87.32	92.72	34.08	62.56	73.06	70.76

*The table shows originally reported scores (DeVLBERT reported and ViLBERT reported), scores for retrained models (DeVLBERT repro, DeVLBERT-CkptCopy, ViLBERT repro, average over 5 runs), and scores for adapted models (DeVLBERT-DepPrior, DeVLBERT-NoPrior). The color scaling (darker for higher score) is per column*.

We make a couple of observations:

The top two rows show the improvement of DeVLBERT over ViLBERT as reported in Zhang et al. (2020b). The next two rows show the same comparison, but for our like-for-like reproduction where we retrained DeVLBERT and ViLBERT from scratch. Contrary to Zhang et al. (2020b) we do *not* observe an improvement across the downstream tasks. Part of the gap is closed by the better score of ViLBERT. Especially on IR and ZSIR, we see that training for more epochs improves the R@1 for ViLBERT by almost 2 to 3 percentage points. This indicates that the reported improvement in Zhang et al. (2020b) might be largely due to differences in training, rather than due to *AutoDeconfounding*.

However, another part of the difference between the reported scores and our reproduces scores is that we get a lower score than reported for DeVLBERT in our reproductions. As we use the code provided by Zhang et al. (2020b) to retrain models, we hypothesize that this difference is due to the slightly smaller size of the pretraining dataset that was available to us.

We see that DeVLBERT-CkptCopy scores are different from the reported scores. This is because the model checkpoint that the authors of DeVLBERT made available is not the one for which they reported results^19^.

Although we could not quite reproduce the results in the same way, our results indicate that the reported improvement in Zhang et al. (2020b) might be mainly due to a different training regime.

Finally, the results for DeVLBERT-DepPrior and DeVLBERT-NoPrior do not show a consistent degradation of performance on all downstream tasks when compared to DeVLBERT repro. This indicates that the $P_{Z}$ component of *AutoDeconfounding* indeed is not a key component.

All in all, these results cast serious doubt on the validity of *AutoDeconfounding* as a method to improve performance on out-of-domain downstream tasks.

### 6.2. Investigating Confounder Finding

#### 6.2.1. Quantitative Analysis

##### Setup

For the experiments that investigate confounder finding, we only evaluate runs that are variations of DeVLBERT, but not runs that are variations of ViLBERT. ViLBERT does not contain the mechanism that selects confounders, shown in Equation (21), and so cannot be judged on whether it finds confounders. More specifically, we evaluate DeVLBERT repro, DeVLBERT-CkptCopy, DeVLBERT-DepPrior, and DeVLBERT-NoPrior.

##### Results

Table 5 shows the mAP results.

**Table 5:** Comparison of confounder-finding performance of DeVLBERT with a random baseline.

	**mAP score**	**mAP excess over random baseline**
**Run name**		
DeVLBERT repro (5 run avg ± stdev)	0.81 ± 0.03	0.1 ± 0.03
DeVLBERT-CkptCopy	0.68	-0.02
DeVLBERT-DepPrior	0.65	-0.05
DeVLBERT-NoPrior	0.80	0.10

*Recall from section 5.3.1.3 that the mAP score is calculated based on how the attention weights rank candidate causes for which we have a ground-truth label in the self-collected dataset described in section 5.3.1.2*.

Table 5 shows that the reproduced runs behave differently than DeVLBERT-CkptCopy. Whereas DeVLBERT-CkptCopy is worse than random at correctly ranking the causes in the ground truth dataset, the reproduced runs score better than random. We trained multiple reproductions to see whether this was due to high variance for the mAP score over different initializations, but this behavior holds up over 5 runs. Recall from section 6.1 that the reproduced model scores slightly *lower* than DeVLBERT-CkptCopy on downstream tasks. This indicates that finding or not finding confounders does not correlate with performance on the downstream tasks.

Further, for DeVLBERT-DepPrior and DeVLBERT-NoPrior, we get mixed results. DeVLBERT-NoPrior shows a similar result to the reproduced runs, while DeVLBERT-DepPrior again shows a worse-than-random result. Given the Causal Hierarchy Theorem mentioned in section 2, we might expect that it would not be possible to correctly identify causes given only observational data, and so we would expect all runs to score around the random baseline. We propose the following explanation for this apparent paradox. While the CHT states that the correct SCM cannot be recovered from only observational data, it *can* be recovered up to its Markov Equivalence Class. We hypothesize that for the ranking task we developed, this might be sufficient to score above-random. We hypothesize that a more specialized ranking task that explicitly seeks to test cause-finding beyond what can be deduced up to the level of a Markov Equivalence Class will be harder. Performance on such a harder confounder-finding task might then be a better predictor of out-of-distribution performance. We leave the development of such a task for future work.

In conclusion, we get mixed results in trying to evaluate ground truth confounder-finding, however the results do indicate that confounder-finding ability does not correlate with performance on downstream tasks.

#### 6.2.2. Qualitative Analysis

##### Setup

For the qualitative investigation of confounder-finding, we do not average the result of DeVLBERT repro, but show the result for only one of the runs. Because different runs might produce a different ranking, it is hard to display the average results in a compact way. Because it is a qualitative investigation, the result for a single run is sufficiently informative. We display the run with the best mAP score.

##### Results

The full 1600 × 1600 tables with the attention distributions can be found in the Supplementary Material. The scores shown are the values of α[*c*] in Equation (21), averaged over all records containing the effect variable. Table 6 shows a subset, that is, the top ranked classes for the 10 most common objects.

**Table 6:** The average top predicted “causes” in the confounder dictionary Z and the corresponding average attention score α[*c*] for the 10 most common objects (in bold in the leftmost column).

	**Effect variable**	**Top cause variables**			
DeVLBERT repro (best run)	Man	key: 0.029	brick: 0.028	kitchen: 0.019	houses: 0.019
	Building	grape: 0.054	strawberry: 0.033	skull: 0.032	brick: 0.03
	Woman	key: 0.03	brick: 0.029	kitchen: 0.022	houses: 0.019
	Tree	skull: 0.043	key: 0.035	grape: 0.031	brick: 0.027
	Window	brick: 0.031	houses: 0.022	strawberry: 0.022	grape: 0.018
	Shirt	cupcake: 0.026	skull: 0.024	houses: 0.021	kitchen: 0.019
	Sky	cupcake: 0.029	grape: 0.026	skull: 0.023	brick: 0.021
	Wall	key: 0.038	grape: 0.034	brick: 0.028	strawberry: 0.023
	Hair	key: 0.036	kitchen: 0.026	restaurant: 0.023	sunset: 0.02
	Head	key: 0.03	strawberry: 0.022	kitchen: 0.021	restaurant: 0.02
DeVLBERT-CkptCopy	Man	butter knife: 0.943	home: 0.001	little girl: 0.0	strawberry: 0.0
	Building	butter knife: 0.961	home: 0.001	little girl: 0.0	doll: 0.0
	Woman	butter knife: 0.937	home: 0.001	little girl: 0.001	flooring: 0.0
	Tree	butter knife: 0.948	home: 0.001	strawberry: 0.0	orange: 0.0
	Window	butter knife: 0.957	home: 0.001	strawberry: 0.0	flooring: 0.0
	Shirt	butter knife: 0.935	home: 0.001	little girl: 0.0	skull: 0.0
	Sky	butter knife: 0.965	home: 0.001	grape: 0.0	flooring: 0.0
	Wall	butter knife: 0.969	home: 0.001	flooring: 0.0	doll: 0.0
	Hair	butter knife: 0.909	home: 0.001	little girl: 0.001	grape: 0.001
	Head	butter knife: 0.917	little girl: 0.001	home: 0.001	strawberry: 0.001
DeVLBERT-NoPrior	Man	floret: 0.013	pizzas: 0.013	remotes: 0.012	products: 0.011
	Building	cub: 0.015	veggie: 0.014	floret: 0.013	boulders: 0.012
	Woman	floret: 0.014	pizzas: 0.012	control: 0.012	pizza slice: 0.011
	Tree	floret: 0.017	cub: 0.016	boulders: 0.014	products: 0.012
	Window	cub: 0.017	boulders: 0.016	remotes: 0.012	floret: 0.012
	Shirt	control: 0.013	pizzas: 0.012	floret: 0.012	air vent: 0.011
	Sky	lunch: 0.014	cub: 0.013	products: 0.012	pizzas: 0.012
	Wall	cub: 0.015	pizzas: 0.012	key: 0.011	trick: 0.011
	Hair	floret: 0.015	remotes: 0.014	motorcyclist: 0.013	bath tub: 0.013
	Head	remotes: 0.014	floret: 0.013	bath tub: 0.013	motorcyclist: 0.012
DeVLBERT-DepPrior	Man	sun: 0.018	palm tree: 0.012	star: 0.012	balloon: 0.012
	Building	balloon: 0.023	thumb: 0.018	triangle: 0.015	elephant: 0.013
	Woman	apple: 0.018	palm tree: 0.013	vehicle: 0.013	newspaper: 0.012
	Tree	balloon: 0.031	brick: 0.018	paint: 0.014	stone: 0.011
	Window	dot: 0.018	hole: 0.016	button: 0.015	bolt: 0.012
	Shirt	sheep: 0.027	border: 0.021	hay: 0.018	skull: 0.016
	Sky	elephant: 0.015	cake: 0.013	balloon: 0.012	thumb: 0.012
	Wall	rain: 0.011	sheep: 0.01	moon: 0.009	string: 0.009
	Hair	pavement: 0.019	rain: 0.016	blanket: 0.015	vehicle: 0.015
	Head	pavement: 0.02	vehicle: 0.018	balloon: 0.016	apple: 0.014

Table 7 shows the same values, but for a particular example of each effect variable rather than for the average^20^.

**Table 7:** The top predicted “causes” in the confounder dictionary Z and the corresponding attention score α[*c*], not averaged over all images as in Table 6, but for the objects detected in the specific images shown above.

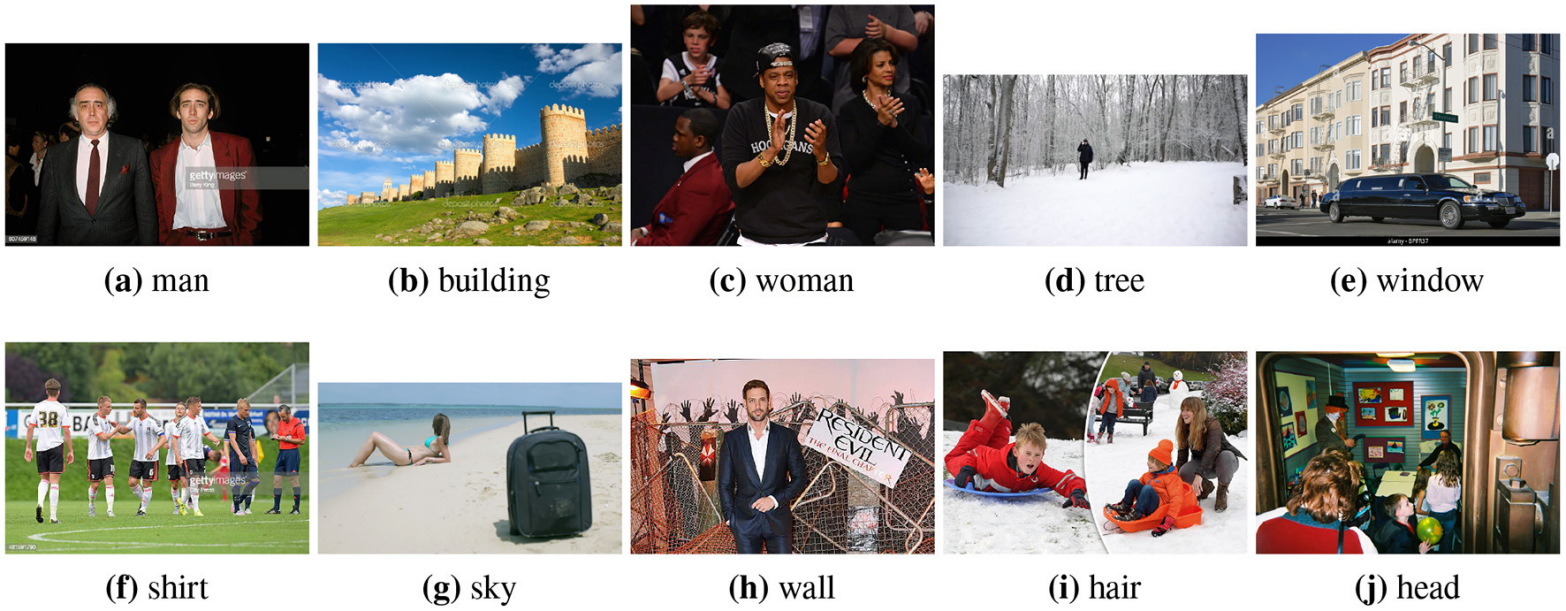					
	**Effect variable**	**Top cause variables**			
DeVLBERT repro (best run)	Man	key: 0.03	brick: 0.027	orange: 0.025	kitchen: 0.022
	Building	key: 0.012	strawberry: 0.011	skull: 0.01	brick: 0.009
	Woman	key: 0.032	kitchen: 0.03	brick: 0.029	apple: 0.023
	Tree	key: 0.05	skull: 0.048	grape: 0.028	strawberry: 0.027
	Window	brick: 0.031	houses: 0.023	tile: 0.022	skull: 0.021
	Shirt	cupcake: 0.034	houses: 0.026	skull: 0.025	pumpkin: 0.025
	Sky	cupcake: 0.047	grape: 0.046	kites: 0.036	mouse pad: 0.027
	Wall	cupcake: 0.039	key: 0.037	grape: 0.028	brick: 0.028
	Hair	key: 0.043	kitchen: 0.028	sunset: 0.028	cupcake: 0.026
	Head	key: 0.025	cupcake: 0.024	page: 0.022	brick: 0.021
DeVLBERT-CkptCopy	Man	butter knife: 1.0	flooring: 0.0	home: 0.0	skull: 0.0
	Building	butter knife: 1.0	home: 0.0	wild: 0.0	houses: 0.0
	Woman	butter knife: 0.999	little girl: 0.0	skull: 0.0	flooring: 0.0
	Tree	butter knife: 0.999	home: 0.0	orange: 0.0	girls: 0.0
	Window	butter knife: 0.999	home: 0.0	adult: 0.0	flooring: 0.0
	Shirt	butter knife: 1.0	home: 0.0	grape: 0.0	skull: 0.0
	Sky	butter knife: 0.962	flooring: 0.001	home: 0.001	adult: 0.0
	Wall	butter knife: 1.0	end table: 0.0	little girl: 0.0	flooring: 0.0
	Hair	butter knife: 0.994	grape: 0.0	blueberry: 0.0	strawberry: 0.0
	Head	butter knife: 1.0	wild: 0.0	home: 0.0	grape: 0.0
DeVLBERT-NoPrior	Man	lunch: 0.021	ox: 0.02	meal: 0.017	racer: 0.017
	Building	trick: 0.024	home: 0.023	pizza slice: 0.019	cub: 0.018
	Woman	sign post: 0.004	pizza slice: 0.004	pizzas: 0.004	breast: 0.004
	Tree	sandwiches: 0.009	wild: 0.007	home: 0.006	book shelf: 0.006
	Window	windshield wipers: 0.031	foliage: 0.024	plain: 0.024	lunch: 0.022
	Shirt	mountain range: 0.024	windshield wipers: 0.022	trick: 0.021	electrical outlet: 0.019
	Sky	lunch: 0.028	trick: 0.024	seasoning: 0.023	pizza slice: 0.019
	Wall	trick: 0.025	jets: 0.022	lunch: 0.019	plain: 0.015
	Hair	pizzas: 0.025	soil: 0.024	windshield wipers: 0.023	lunch: 0.023
	Head	windshield wipers: 0.031	flooring: 0.021	mountain range: 0.02	pizzas: 0.018
DeVLBERT-DepPrior	Man	palm tree: 0.028	graffiti: 0.026	branches: 0.026	photo: 0.023
	Building	moon: 0.085	bolt: 0.05	hole: 0.045	dot: 0.039
	Woman	skull: 0.076	string: 0.031	sheep: 0.022	graffiti: 0.021
	Tree	string: 0.046	board: 0.036	newspaper: 0.034	bolt: 0.03
	Window	balloon: 0.061	button: 0.046	dot: 0.035	newspaper: 0.034
	Shirt	skull: 0.038	star: 0.024	light: 0.022	border: 0.022
	Sky	bench: 0.011	baby: 0.008	plane: 0.007	flower: 0.007
	Wall	string: 0.034	balloon: 0.032	sheep: 0.022	hay: 0.017
	Hair	umbrella: 0.054	skull: 0.049	screen: 0.042	horse: 0.031
	Head	skull: 0.028	mouse: 0.028	word: 0.024	candle: 0.023

For DeVLBERT-CkptCopy, the by-far most-attended-to element of the confounder dictionary Z is “butter knife,” and the rest of top-attended elements are similar between objects. For the other runs, there is no one most-attended-to element, but we also see a small set of classes appearing as top-cause candidates for different effect variables.

To explain the high-confidence “butter knife”-selecting behavior, we hypothesize that DeVLBERT-CkptCopy found a beneficial local optimum that makes use of Z in a specific way. The low-confidence predictions for the other runs then indicate that they did not find such an optimum.

It does *not* seem to be the case however that either case corresponds to finding actual confounders. For DeVLBERT-CkptCopy, it seems unlikely that “butter knife” is indeed the most likely confounder for every class. The top causes selected by the other models also do not seem intuitively causal: “key” causing “man” for DeVLBERT repro, “cub” causing “building” for DeVLBERT-NoPrior, or “apple” causing “woman” for DeVLBERT-DepPrior do not seem related to true causality.

The fact that the same classes appear as top causes for unrelated effect variables indicates an indifference of the model to the value of the effect variable: Which cause variable receives a large attention score, depends more on the value of the particular (fixed) embedding for that cause variable, than on how well it matches with the effect variable for which a cause is supposedly found. Note that the cause variable representation comes from the (fixed) confounder dictionary, whereas the effect variable representation is the contextualized output of the Transformer model. We hypothesize that this asymmetry is due to the projection matrices that precede the inner product in the calculation of attention scores. The model might have found it beneficial to make the weights in these projection matrices such that the attention output is more related to the cause variable than to the effect variable.

Note that the attention parameters in *AutoDeconfounding* are never explicitly trained to make confounder-finding predictions. Rather, it is assumed by *AutoDeconfounding* that the attention scores can be interpreted as cause-selecting-scores. The selection of top causes observed in Table 6 indicates that this interpretation is not valid.

Table 8 shows a few example images of a downstream task (VQA), where the query word is an effect, and the bounding box shown is that of a cause. If a model learned to depend less on spurious correlations and more on causes during pretraining, it is expected that this is reflected in higher attention values from effects to causes. We do not observe that the models using variations of *AutoDeconfounding* pay significantly more attention to the cause object than the baseline ViLBERT model.

**Table 8:** Some examples of the attention from query word to cause bounding box, for different models.

**VQA Question**	**Is there a car on the road?**	**What is odd about the dog's eyes**	**What color is the person's helmet?**
Object in bounding box	Street	Head	Person
DeVLBERT	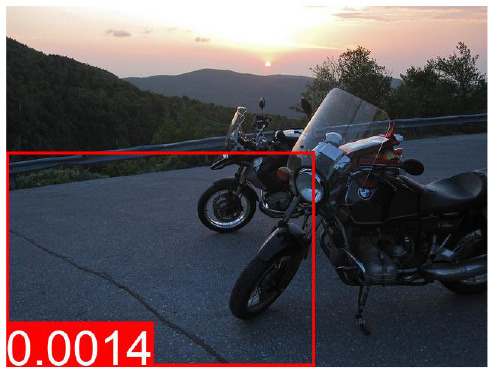	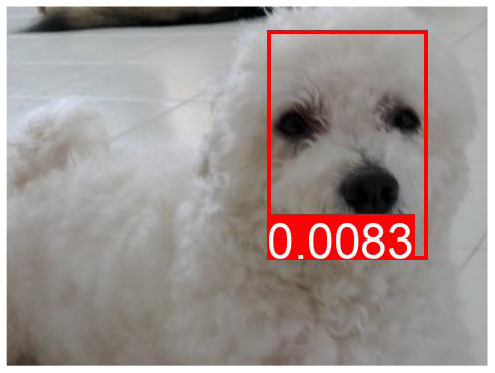	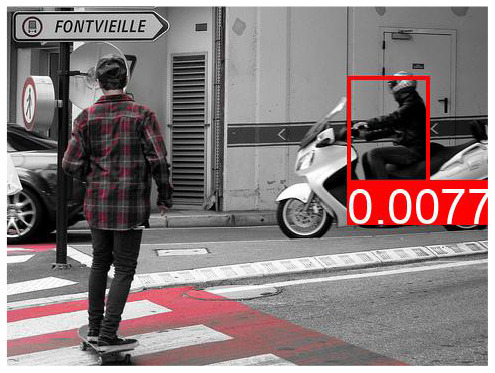
ViLBERT	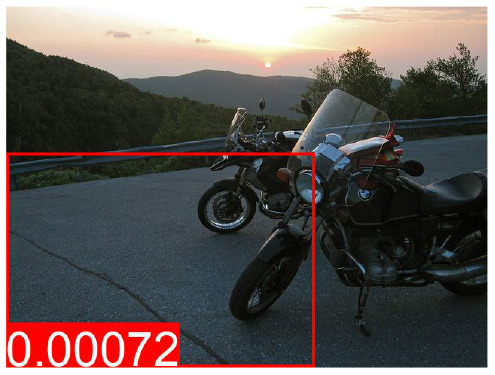	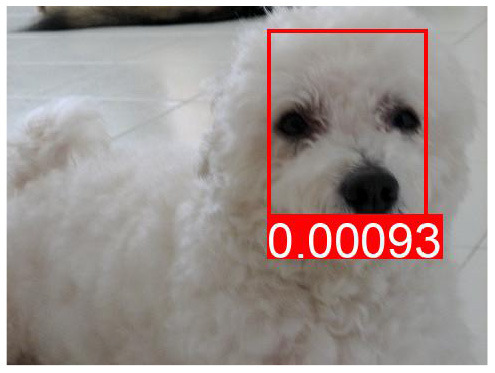	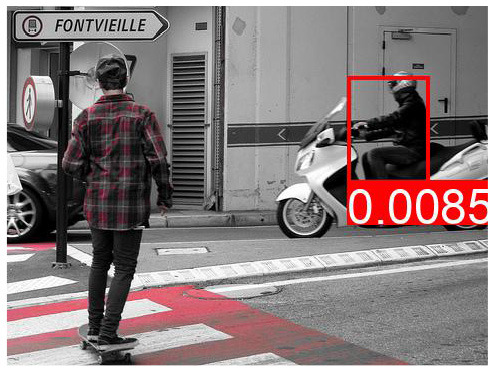
DeVLBERT-NoPrior	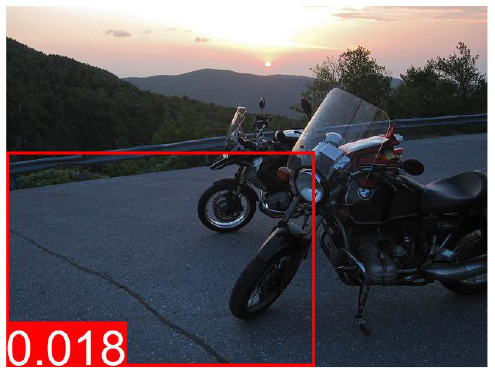	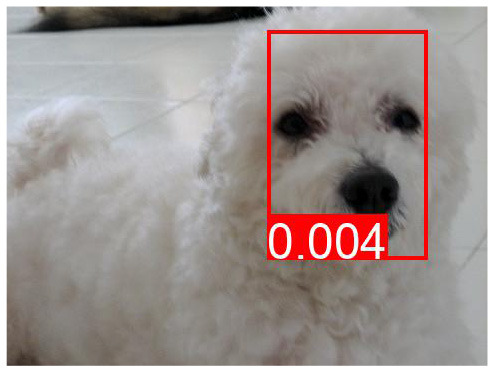	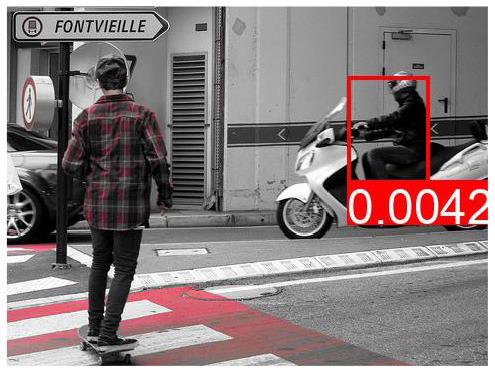
DeVLBERT-DepPrior	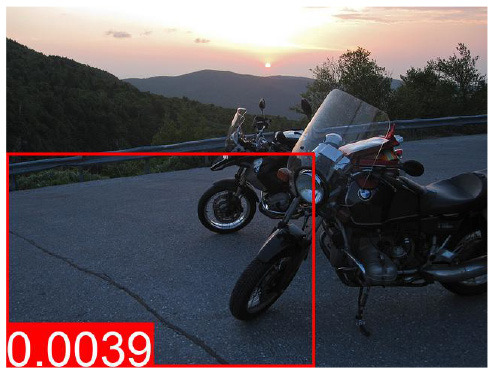	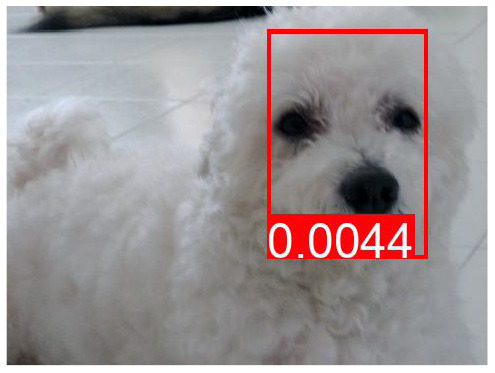	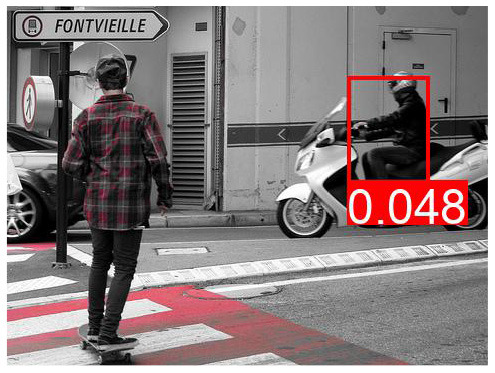

*The word colored red in the VQA question is the query for the attention. Out of all possible bounding boxes to display, a bounding box with a “cause” for the query is manually selected, and displayed. Note that it is not necessary for the query word to be present in the image. For the presence of “car,” presence of “street” is a cause, for the presence of ‘eyes,” presence of “head” is a cause, and for the presence of “helmet,” presence of “person” is a cause. The number on the image indicates the attention score given by the model to that bounding box*.

## 7. Conclusion

Models relying on spurious correlations are an important issue, and leveraging causal knowledge to address the issue is a promising approach. Causal models benefit transfer learning, allowing for faster adaptation to other distributions and thus being more generalizable (Schölkopf, 2019). This has been a popular approach to tackle spurious correlations specifically in the visio-linguistic domain, where a number of works have used it to further improve on the already impressive representation-learning capacities of Transformer-like models.

Leveraging causality with *automatically* discovered causal structure is especially interesting, as it could scale much better than human-labeled causal structure. This critical analysis has uncovered some of the issues with this approach.

First, care needs to be taken in being specific with regard to the underlying causal model that is assumed. As shown in section 5.1, only when making the link between causal variables and data-representations explicit is it possible to specify the assumptions under which an implementation of deconfounding is valid. An interesting avenue for future work could be to adapt the implementation to work with a less strict set of assumptions. Furthermore, more thought should be given to the design of models that produce interpretable representations that provide insight in the causal structure and relations of objects captured by these models. It would also be insightful to validate whether the assumptions hold for the application of interest.

Second, it is important to *isolate* the effect of causal representation learning: as section 5.2 shows, when doing a like-for-like comparison, in which the baseline is reproduced under the same circumstances, it is important to avoid confusing the effect of training circumstances with those of the added loss. Moreover, it is crucial to ablate every component to verify to what extent it is responsible for the improvement of the whole. Specifically for out-of-distribution applications, it would be interesting to discover which spurious correlations change between the distributions of interest, and whether those have been correctly captured by the model.

Finally, a key element in leveraging causality with automatically discovered causal structure is assessing to what extent the discovered structure is accurate. Our investigation using human-proxy-for-ground-truth shows mixed results in this regard, with the models that perform better on downstream visio-linguistic tasks scoring worse than random in a cause-ranking task. First testing models on domains for which the causal structure is known, for instance, as done by Lopez-Paz et al. (2017) can help to build confidence in the fact that causal operations such as deconfounding are realized using the correct causal model.

For future work, creating more extensive causally annotated datasets can enable progress in causal discovery. Additionally, it can be interesting to explore causal models that are more fine-grained than object co-occurrence, as spurious correlations are present at the level of objects as well as the level of object attributes. For example, taking attributes as causal variables, or making use of temporal data such as videos. More fine-grained variables can also be expected to be useful for novel distributions with unseen objects: if the unseen objects consist of known “parts,” their causal properties could still be predicted.

## Data Availability Statement

The datasets presented in this study can be found in online repositories. The names of the repository/repositories and accession number(s) can be found in the article/Supplementary Material.

## Author Contributions

NC came up with the idea to critically investigate *AutoDeconfounding*, conducted the experiments, created the figures, and was the main contributor to the text. KL and M-FM provided useful insights and suggestions during meetings, and provided suggestions and corrections for this article text during writing. All authors contributed to the article and approved the submitted version.

## Funding

This research was financed by the CALCULUS project—Commonsense and Anticipation enriched Learning of Continuous representations—European Research Council Advanced Grant H2020-ERC-2017-ADG 788506, http://calculus-project.eu/. KL was supported by a grant of the Research Foundation—Flanders (FWO) no. 1S55420N.

## Conflict of Interest

The authors declare that the research was conducted in the absence of any commercial or financial relationships that could be construed as a potential conflict of interest.

## Publisher's Note

All claims expressed in this article are solely those of the authors and do not necessarily represent those of their affiliated organizations, or those of the publisher, the editors and the reviewers. Any product that may be evaluated in this article, or claim that may be made by its manufacturer, is not guaranteed or endorsed by the publisher.

## Footnotes

^1^Intuitively, intervening on the value of some variable (like the presence of an object in a scene) means manipulating its value independently of the value of all other variables. This is defined formally in section 3.3.

^2^For example, the correlation observed between umbrellas in the street, and number of taxis being taken is not due to a direct causal link, but due to the common cause of rainy weather.

^3^Also Structural Equation Model.

^4^The set of SCMs that encode the same set of conditional probabilities.

^5^Note that the way $P_{Z}$ is normalized implies that if every class would appear in every image, each class would have a value of $\frac{1}{C}$ in $P_{Z}$ rather than a value of 1.

^6^Note that because a softmax is used to calculate the attention score, it is not possible to give more than one confounder a weight of 1.

^7^Up-Down (Anderson et al., 2018) for captioning and VQA, AoANet (Huang et al., 2019) for captioning, MCAN (Yu et al., 2019) for VQA, R2C (Zellers et al., 2019) and ViLBERT (Lu et al., 2019) for VCR.

^8^See DeVLBERT replication instructions at https://github.com/shengyuzhang/DeVLBert.

^9^“Almost-all” is meant in a measure-theoretic sense, explained in Bareinboim et al. (2020).

^10^Although correlation is not a necessary condition for causation, for this heuristic we assume this is still a useful way to weed out many uninteresting pairwise relations.

^11^We do not use the absolute value of *log*(*P*(*X* = 1, *Y* = 1)/*P*(*X* = 1)*P*(*Y* = 1)) because this fills the top of the ranking with pairs of classes that occur only once but not together: for these pairs, *log*(*P*(*X* = 1, *Y* = 1)/*P*(*X* = 1)*P*(*Y* = 1)) is minus infinity. These pairs are almost all unrelated and thus not causally meaningful.

^12^The absolute difference surfaces more common classes, while the relative difference also surfaces rare classes.

^13^We detail the crowdworkers' reward per label and estimated time spent per label in the Ethical Considerations section at the end of this article.

^14^We did not end up weighting agreement by confidence level as the difference in resulting pairs was small anyway.

^15^ https://drive.google.com/file/d/17CTPMoZ4uJH6cSQaxD6Vmyv_bVLJwVJp/view?usp=sharing

^16^For pretraining, the batch size is 512, for finetuning VQA, it is 256, and for finetuning IR it is 64.

^17^Using 8 32GB NVIDIA V100 GPUs with a batch size of 64 per GPU, training takes 3 days. Using 4 16GB NVIDIA P100 GPUS with batch size 64 and gradient accumulation of 2 (making an effective batch size 512), training takes about 5 days.

^18^The code to reproduce all results can be found on github: https://github.com/Natithan/p1_causality.

^19^This was confirmed after correspondence with the authors of DeVLBERT.

^20^The original images for “woman” and “wall” have a high height/width ration, and are shown trimmed for display purposes.
