# Supplementary material for: Critical Analysis of Deconfounded Pretraining to Improve Visio-Linguistic Models
Source: Front Artif Intell. 2022 Mar 17;5:736791. doi: 10.3389/frai.2022.736791 (PMC8993511; doi:10.3389/frai.2022.736791)
Supplement: Supplementary file 1 [file Presentation_1.pdf]

Critical Analysis of Deconfounded Pretraining to Improve Visio-Linguistic Models

Nathan Cornille [1,*], **Katrien Laenen** [1] **and Marie-Francine Moens** [1]

[1] *LIIR, KU Leuven, Department of Computer Science, Leuven, Belgium*

Correspondence*:
Nathan Cornille
nathan.cornille@kuleuven.be

This is the publicly accessible link to the google drive folder with all supplementary material: `https://drive.google.com/drive/folders/1NO8P8r4pTgarsQdlwjp7TAT5T4MtSotC?usp=sharing`

Figure 1. Example of form provided to crowdworkers. An html file of the full page can be found at `https://drive.google.com/file/d/1Zhx54OLY1ijpXbsCzsTY4mwSpa7FjV1g/view?usp=sharing`

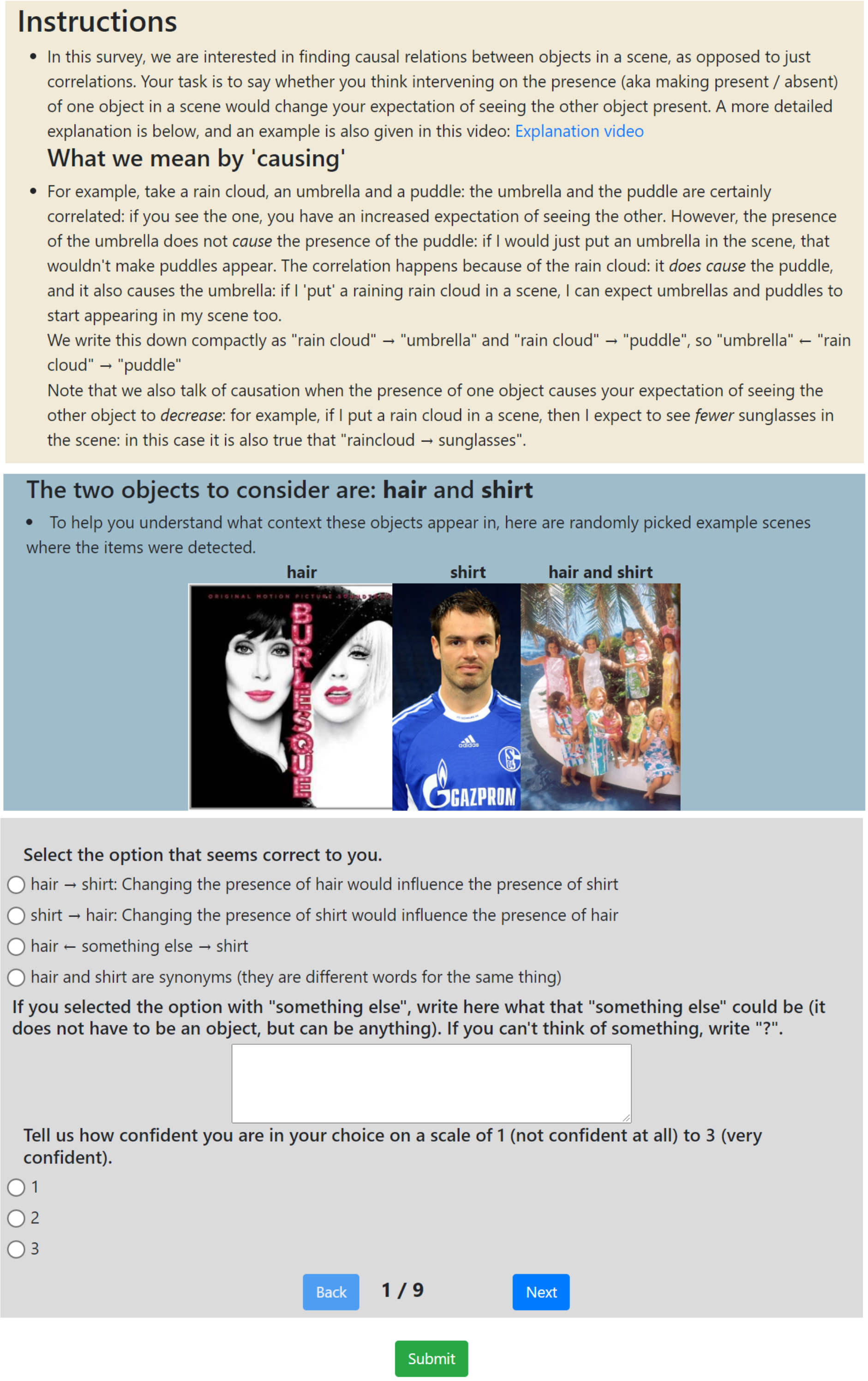

Instructions

- In this survey, we are interested in finding causal relations between objects in a scene, as opposed to just correlations. Your task is to say whether you think intervening on the presence (aka making present / absent) of one object in a scene would change your expectation of seeing the other object present. A more detailed explanation is below, and an example is also given in this video: Explanation video

What we mean by 'causing'

- For example, take a rain cloud, an umbrella and a puddle: the umbrella and the puddle are certainly correlated: if you see the one, you have an increased expectation of seeing the other. However, the presence of the umbrella does not *cause* the presence of the puddle: if I would just put an umbrella in the scene, that wouldn't make puddles appear. The correlation happens because of the rain cloud: it *does cause* the puddle, and it also causes the umbrella: if I 'put' a raining rain cloud in a scene, I can expect umbrellas and puddles to start appearing in my scene too.
  We write this down compactly as "rain cloud" → "umbrella" and "rain cloud" → "puddle", so "umbrella" ← "rain cloud" → "puddle"
  Note that we also talk of causation when the presence of one object causes your expectation of seeing the other object to *decrease*: for example, if I put a rain cloud in a scene, then I expect to see *fewer* sunglasses in the scene: in this case it is also true that "raincloud → sunglasses".

The two objects to consider are: **hair** and **shirt**

- To help you understand what context these objects appear in, here are randomly picked example scenes where the items were detected.

hair **shirt** **hair and shirt**

Select the option that seems correct to you.

- ○ hair → shirt: Changing the presence of hair would influence the presence of shirt
- ○ shirt → hair: Changing the presence of shirt would influence the presence of hair
- ○ hair ← something else → shirt
- ○ hair and shirt are synonyms (they are different words for the same thing)

If you selected the option with "something else", write here what that "something else" could be (it does not have to be an object, but can be anything). If you can't think of something, write "?".

Tell us how confident you are in your choice on a scale of 1 (not confident at all) to 3 (very confident).

- ○ 1
- ○ 2
- ○ 3

Back **1 / 9** Next

Submit

Figure 2. Detail of the test on which crowdworkers had to score 90% or more. An html file with the full page can be found at `https://drive.google.com/file/d/1Zhx54OLY1ijpXbsCzsTY4mwSpa7FjV1g/view?usp=sharing`

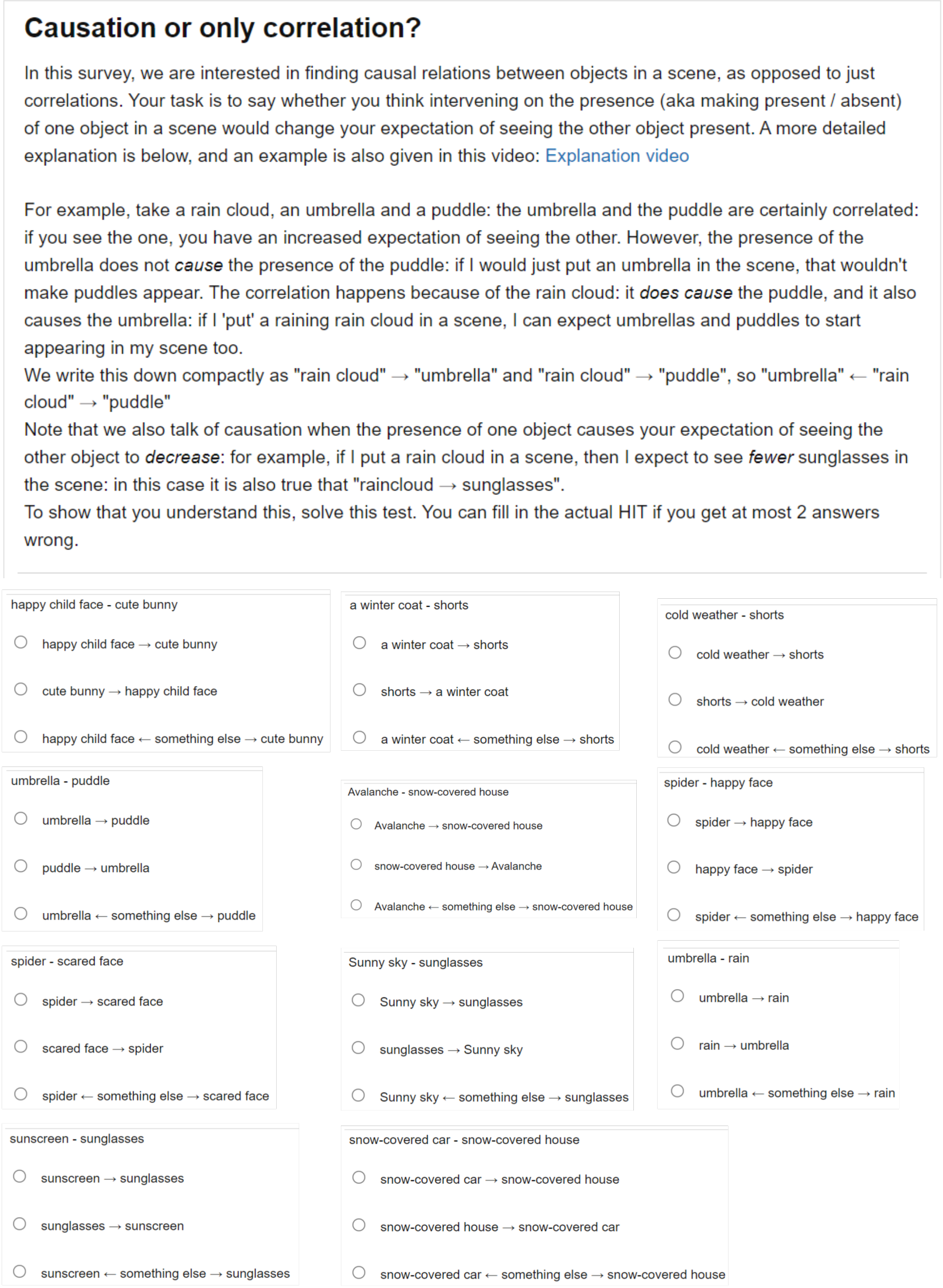

Causation or only correlation?

In this survey, we are interested in finding causal relations between objects in a scene, as opposed to just correlations. Your task is to say whether you think intervening on the presence (aka making present / absent) of one object in a scene would change your expectation of seeing the other object present. A more detailed explanation is below, and an example is also given in this video: Explanation video

For example, take a rain cloud, an umbrella and a puddle: the umbrella and the puddle are certainly correlated: if you see the one, you have an increased expectation of seeing the other. However, the presence of the umbrella does not *cause* the presence of the puddle: if I would just put an umbrella in the scene, that wouldn't make puddles appear. The correlation happens because of the rain cloud: it *does cause* the puddle, and it also causes the umbrella: if I 'put' a raining rain cloud in a scene, I can expect umbrellas and puddles to start appearing in my scene too.
We write this down compactly as "rain cloud" → "umbrella" and "rain cloud" → "puddle", so "umbrella" ← "rain cloud" → "puddle"
Note that we also talk of causation when the presence of one object causes your expectation of seeing the other object to *decrease*: for example, if I put a rain cloud in a scene, then I expect to see *fewer* sunglasses in the scene: in this case it is also true that "raincloud → sunglasses".
To show that you understand this, solve this test. You can fill in the actual HIT if you get at most 2 answers wrong.

happy child face - cute bunny
- happy child face → cute bunny
- cute bunny → happy child face
- happy child face ← something else → cute bunny

a winter coat - shorts
- a winter coat → shorts
- shorts → a winter coat
- a winter coat ← something else → shorts

cold weather - shorts
- cold weather → shorts
- shorts → cold weather
- cold weather ← something else → shorts

umbrella - puddle
- umbrella → puddle
- puddle → umbrella
- umbrella ← something else → puddle

Avalanche - snow-covered house
- Avalanche → snow-covered house
- snow-covered house → Avalanche
- Avalanche ← something else → snow-covered house

spider - happy face
- spider → happy face
- happy face → spider
- spider ← something else → happy face

spider - scared face
- spider → scared face
- scared face → spider
- spider ← something else → scared face

Sunny sky - sunglasses
- Sunny sky → sunglasses
- sunglasses → Sunny sky
- Sunny sky ← something else → sunglasses

umbrella - rain
- umbrella → rain
- rain → umbrella
- umbrella ← something else → rain

sunscreen - sunglasses
- sunscreen → sunglasses
- sunglasses → sunscreen
- sunscreen ← something else → sunglasses

snow-covered car - snow-covered house
- snow-covered car → snow-covered house
- snow-covered house → snow-covered car
- snow-covered car ← something else → snow-covered house

PROOF OF EQUIVALENCE BETWEEN *AUTODECONFOUNDING* AND DECONFOUNDING UNDER CERTAIN ASSUMPTIONS.

For ease of notation, we introduce $\mathcal{ZC}$ and $\mathcal{P}_{\mathcal{ZC}}$:

$$\mathcal{ZC} = concat[\mathcal{Z}[i] \text{ for i } \in S_z] \tag{1}$$

$$\mathcal{P}_{\mathcal{ZC}} = concat[\mathcal{P}_{\mathcal{Z}}[i] \text{ for i } \in S_z] \tag{2}$$

$$\tag{3}$$

For ease of reference, we duplicate the relevant equations from the main paper here:

$$P(Y = v_y|do(X = v_x)) = \text{softmax}(g(\boldsymbol{f_x}, \sum_{v_{z_1}=0,\ldots,v_{z_c}=0}^{1,\ldots,1} \boldsymbol{f_z} P(Z_1 = v_{z_1}, \ldots, Z_c = v_{z_c})), \tag{4}$$

$$\text{VC-R-CNN: } \boldsymbol{p_y^x} = \text{softmax}(g_V(\boldsymbol{f_x}, \frac{\sum_{i \in S_z} \mathcal{Z}[i]\mathcal{P}_{\mathcal{Z}}[i]}{c}])) \tag{5}$$

$$\text{DeVLBERT: } \boldsymbol{p_y^a} = \text{softmax}(g_D(\frac{\sum_{i \in S_z} \mathcal{Z}^b[i]\mathcal{P}_{\mathcal{Z}}{}^b[i]}{c}])) \tag{6}$$

Then, equation 5 and 6 become:

$$\text{VC-R-CNN: } \boldsymbol{p_y^x} = \text{softmax}(g_V(\boldsymbol{f_x}, \frac{\sum_{i=1}^{c} \mathcal{ZC}[i]\mathcal{P}_{\mathcal{ZC}}[i]]}{c})) \tag{7}$$

$$\text{DeVLBERT: } \boldsymbol{p_y^a} = \text{softmax}(g_D(\frac{\sum_{i=1}^{c} \mathcal{ZC}^b[i]\mathcal{P}_{\mathcal{ZC}}{}^b[i]]}{c})) \tag{8}$$

where c is the number of elements in S_z, a can be either modality t or v, and b can be either modality t or v.

If we assume that all the confounders are independent, that is $P(Z_1 = v_{z_1}, \ldots, Z_c = v_{z_c}) = \prod_{i=1}^{c} P(Z_i = v_{z_i})$, we can link each factor to $\mathcal{P}_{\mathcal{ZC}}$: $P(Z_i = 1)$ corresponds to $\frac{R}{I} \cdot \mathcal{P}_{\mathcal{ZC}}[i]$, where R is the total number of tokens over all records, and I is the total number of records. [1] $P(Z_i = 0)$ then corresponds to $1 - \frac{R}{I} \cdot \mathcal{P}_{\mathcal{ZC}}[i]$.

If we define

$$S_{\mathcal{P}_{\mathcal{ZC}}[i]}(v_{z_i}) = \begin{cases} \frac{R}{I} \cdot \mathcal{P}_{\mathcal{ZC}}[i] & \text{if } v_{z_i} = 1 \\ 1 - \frac{R}{I} \cdot \mathcal{P}_{\mathcal{ZC}}[i] & \text{if } v_{z_i} = 0 \end{cases} \tag{9}$$

Equation 4 then becomes:

$$P(Y = v_y|do(X = v_x)) = \text{softmax}(g(\boldsymbol{f_x}, \sum_{v_{z_1}=0,\ldots,v_{z_c}=0}^{1,\ldots,1} \boldsymbol{f_z} S_{\mathcal{P}_{\mathcal{ZC}}[i]}(v_{z_i}) \ldots S_{\mathcal{P}_{\mathcal{ZC}}[i]}(v_{z_i}))) \tag{10}$$

[1] The constant factor $\frac{R}{I}$ makes up for the fact that the token counts to calculate $\mathcal{P}_{\mathcal{Z}}$ were normalized by dividing by R instead of I.

Because $\boldsymbol{f_z} = \boldsymbol{f_z}(v_1, \ldots, v_c) = \frac{\sum_{i=1}^{c} v_{z_i} \mathcal{ZC}[i]}{c}$, we can write:

$$P(Y = v_y | do(X = v_x)) = \text{softmax}(g(\boldsymbol{f_x}, \sum_{v_{z_1}=0,\ldots,v_{z_c}=0}^{1,\ldots,1} \frac{\sum_{i=1}^{c} v_{z_i} \mathcal{ZC}[i]}{c} S_{\mathcal{P}_{\mathcal{ZC}}[i]}(v_{z_i}) \ldots S_{\mathcal{P}_{\mathcal{ZC}}[i]}(v_{z_i}))) \tag{11}$$

We can show that the second argument of g in equation 11 simplifies to the last argument of g in equations 7 and 8 , in other words that these equations match despite the apparent difference in summation terms:

$$\sum_{v_{z_1}=0,\ldots,v_{z_c}=0}^{1,\ldots,1} \frac{\sum_{i=1}^{c} v_{z_i} \mathcal{ZC}[i]}{c} \prod_{j=1}^{c} S_{\mathcal{P}_{\mathcal{ZC}}[i]}(v_{z_i}) \tag{12}$$

$$\frac{\sum_{i=1}^{c} \mathcal{ZC}[i] \mathcal{P}_{\mathcal{ZC}}[i]}{c} \tag{13}$$

We can derive this equivalence (barring a constant factor) by recursion in the number of confounders c.

First, we show that an auxiliary variable H_x equals 1 for any $x > 0$

$$H_1 = \sum_{v_{z_1}=0,\ldots,v_{z_1}=0}^{1,\ldots,1} \prod_{j=1}^{1} S_{\mathcal{P}_{\mathcal{ZC}}[i]}(v_{z_i}) = S_{\mathcal{P}_{\mathcal{ZC}}[1]}(0) + S_{\mathcal{P}_{\mathcal{ZC}}[1]}(1) = (1 - \frac{R}{I} \cdot \mathcal{P}_{\mathcal{ZC}}[1]) + \frac{R}{I} \cdot \mathcal{P}_{\mathcal{ZC}}[1] = 1 \tag{14}$$

$$H_c = \sum_{v_{z_1}=0,\ldots,v_{z_c}=0}^{1,\ldots,1} \prod_{j=1}^{c} S_{\mathcal{P}_{\mathcal{ZC}}[i]}(v_{z_i}) \tag{15}$$

$$= S_{\mathcal{P}_{\mathcal{ZC}}[c]}(0) \sum_{v_{z_1}=0,\ldots,v_{z_{c-1}}=0}^{1,\ldots,1} \prod_{j=1}^{c-1} S_{\mathcal{P}_{\mathcal{ZC}}[i]}(v_{z_i}) + S_{\mathcal{P}_{\mathcal{ZC}}[c]}(1) \sum_{v_{z_1}=0,\ldots,v_{z_{c-1}}=0}^{1,\ldots,1} \prod_{j=1}^{c-1} S_{\mathcal{P}_{\mathcal{ZC}}[i]}(v_{z_i}) \tag{16}$$

$$= (S_{\mathcal{P}_{\mathcal{ZC}}[c]}(0) + S_{\mathcal{P}_{\mathcal{ZC}}[c]}(1)) \sum_{v_{z_1}=0,\ldots,v_{z_{c-1}}=0}^{1,\ldots,1} \prod_{j=1}^{c-1} S_{\mathcal{P}_{\mathcal{ZC}}[i]}(v_{z_i}) \tag{17}$$

$$= (1 - \frac{R}{I} \cdot \mathcal{P}_{\mathcal{ZC}}[i]) + \frac{R}{I} \cdot \mathcal{P}_{\mathcal{ZC}}[i]) H_{c-1} \tag{18}$$

$$= 1 \tag{19}$$

First, for $c = 1$:

$$T_{c=1} = \sum_{v_{z_1}=0,\dots,v_{z_1}=0}^{1,\dots,1} \frac{\sum_{i=1}^{1} v_{z_i} \mathcal{ZC}[i]}{1} \prod_{j=1}^{1} S_{\mathcal{P}_{\mathcal{ZC}}[j]}(v_{z_j}) \tag{20}$$

$$= \sum_{v_{z_1}=0}^{1} \frac{v_{z_1} \mathcal{ZC}[1]}{1} S_{\mathcal{P}_{\mathcal{ZC}}[1]}(v_{z_1}) \tag{21}$$

$$= \mathcal{ZC}[1] \frac{R}{I} \mathcal{P}_{\mathcal{ZC}}[1] \tag{22}$$

$$= \frac{R}{I} \frac{\sum_{i=1}^{1} \mathcal{ZC}[1] \mathcal{P}_{\mathcal{ZC}}[1]}{1} \tag{23}$$

Then, for the recursive step:

$$T_c = \sum_{v_{z_1}=0,\ldots,v_{z_c}=0}^{1,\ldots,1} \frac{\sum_{i=1}^{c} v_{z_i} \mathcal{ZC}[i]}{c} \prod_{j=1}^{c} S_{\mathcal{P}_{\mathcal{ZC}}[i]}(v_{z_i}) \tag{24}$$

$$=S_{\mathcal{P}_{\mathcal{ZC}}[c]}(0) \sum_{v_{z_1}=0,\ldots,v_{z_{c-1}}=0}^{1,\ldots,1} \frac{\sum_{i=1}^{c-1} v_{z_i} \mathcal{ZC}[i] + 0 \cdot \mathcal{ZC}[c]}{c} \prod_{j=1}^{c-1} S_{\mathcal{P}_{\mathcal{ZC}}[j]}(v_{z_j}) + \tag{25}$$

$$S_{\mathcal{P}_{\mathcal{ZC}}[c]}(1) \sum_{v_{z_1}=0,\ldots,v_{z_{c-1}}=0}^{1,\ldots,1} \frac{\sum_{i=1}^{c-1} v_{z_i} \mathcal{ZC}[i] + 1 \cdot \mathcal{ZC}[c]}{c} \prod_{j=1}^{c-1} S_{\mathcal{P}_{\mathcal{ZC}}[j]}(v_{z_j}) \tag{26}$$

$$=S_{\mathcal{P}_{\mathcal{ZC}}[c]}(0) \sum_{v_{z_1}=0,\ldots,v_{z_{c-1}}=0}^{1,\ldots,1} \frac{\sum_{i=1}^{c-1} v_{z_i} \mathcal{ZC}[i]}{c} \prod_{j=1}^{c-1} S_{\mathcal{P}_{\mathcal{ZC}}[j]}(v_{z_j}) + \tag{27}$$

$$S_{\mathcal{P}_{\mathcal{ZC}}[c]}(1) \sum_{v_{z_1}=0,\ldots,v_{z_{c-1}}=0}^{1,\ldots,1} \frac{\sum_{i=1}^{c-1} v_{z_i} \mathcal{ZC}[i]}{c} \prod_{j=1}^{c-1} S_{\mathcal{P}_{\mathcal{ZC}}[j]}(v_{z_j}) + \tag{28}$$

$$S_{\mathcal{P}_{\mathcal{ZC}}[c]}(1) \sum_{v_{z_1}=0,\ldots,v_{z_{c-1}}=0}^{1,\ldots,1} \frac{\mathcal{ZC}[c]}{c} \prod_{j=1}^{c-1} S_{\mathcal{P}_{\mathcal{ZC}}[j]}(v_{z_j}) \tag{29}$$

$$=S_{\mathcal{P}_{\mathcal{ZC}}[c]}(0) \cdot T_{c-1} \frac{c-1}{c} + S_{\mathcal{P}_{\mathcal{ZC}}[c]}(1) \cdot T_{c-1} \frac{c-1}{c} + \tag{30}$$

$$S_{\mathcal{P}_{\mathcal{ZC}}[c]}(1) \frac{\mathcal{ZC}[c]}{c} \sum_{v_{z_1}=0,\ldots,v_{z_{c-1}}=0}^{1,\ldots,1} \prod_{j=1}^{c-1} S_{\mathcal{P}_{\mathcal{ZC}}[j]}(v_{z_j}) \tag{31}$$

$$=(S_{\mathcal{P}_{\mathcal{ZC}}[c]}(0) + S_{\mathcal{P}_{\mathcal{ZC}}[c]}(1)) \cdot T_{c-1} \frac{c-1}{c} + \tag{32}$$

$$S_{\mathcal{P}_{\mathcal{ZC}}[c]}(1) \frac{\mathcal{ZC}[c]}{c} \cdot H_{c-1} \tag{33}$$

$$=T_{c-1} \frac{c-1}{c} + \frac{R}{I} \cdot \frac{\mathcal{ZC}[c] \mathcal{P}_{\mathcal{ZC}}[c]}{c} \tag{34}$$

$$=\frac{R}{I} \frac{\sum_{i=1}^{c-1} \mathcal{ZC}[c-1] \mathcal{P}_{\mathcal{ZC}}[c-1]}{c-1} \frac{c-1}{c} + \frac{R}{I} \cdot \frac{\mathcal{ZC}[c] \mathcal{P}_{\mathcal{ZC}}[c]}{c} \tag{35}$$

$$=\frac{R}{I} \frac{\sum_{i=1}^{c} \mathcal{ZC}[c] \mathcal{P}_{\mathcal{ZC}}[c]}{c} \tag{36}$$

$$\tag{37}$$
